# MFA-UNet: a vessel segmentation method based on multi-scale feature fusion and attention module

**DOI:** 10.3389/fnins.2023.1249331

**Published:** 2023-11-21

**Authors:** Juan Cao, Jiaran Chen, Yuanyuan Gu, Jinjia Liu

**Affiliations:** ^1^School of Information Science and Engineering, Chongqing Jiaotong University, Chongqing, China; ^2^Ningbo Institute of Materials Technology and Engineering, Chinese Academy of Sciences, Ningbo, China

**Keywords:** vessel segmentation, fundus images, deep neural network, self-attention mechanism, deep supervision

## Abstract

**Introduction:**

The accurate segmentation of retinal vessels is of utmost importance in the diagnosis of retinal diseases. However, the complex vessel structure often leads to poor segmentation performance, particularly in the case of microvessels.

**Methods:**

To address this issue, we propose a vessel segmentation method composed of preprocessing and a multi-scale feature attention network (MFA-UNet). The preprocessing stage involves the application of gamma correction and contrast-limited adaptive histogram equalization to enhance image intensity and vessel contrast. The MFA-UNet incorporates the Multi-scale Fusion Self-Attention Module(MSAM) that adjusts multi-scale features and establishes global dependencies, enabling the network to better preserve microvascular structures. Furthermore, the multi-branch decoding module based on deep supervision (MBDM) replaces the original output layer to achieve targeted segmentation of macrovessels and microvessels. Additionally, a parallel attention mechanism is embedded into the decoder to better exploit multi-scale features in skip paths.

**Results:**

The proposed MFA-UNet yields competitive performance, with dice scores of 82.79/83.51/84.17/78.60/81.75/84.04 and accuracies of 95.71/96.4/96.71/96.81/96.32/97.10 on the DRIVE, STARE, CHASEDB1, HRF, IOSTAR and FIVES datasets, respectively.

**Discussion:**

It is expected to provide reliable segmentation results in clinical diagnosis.

## 1 Introduction

**Figure 1:**
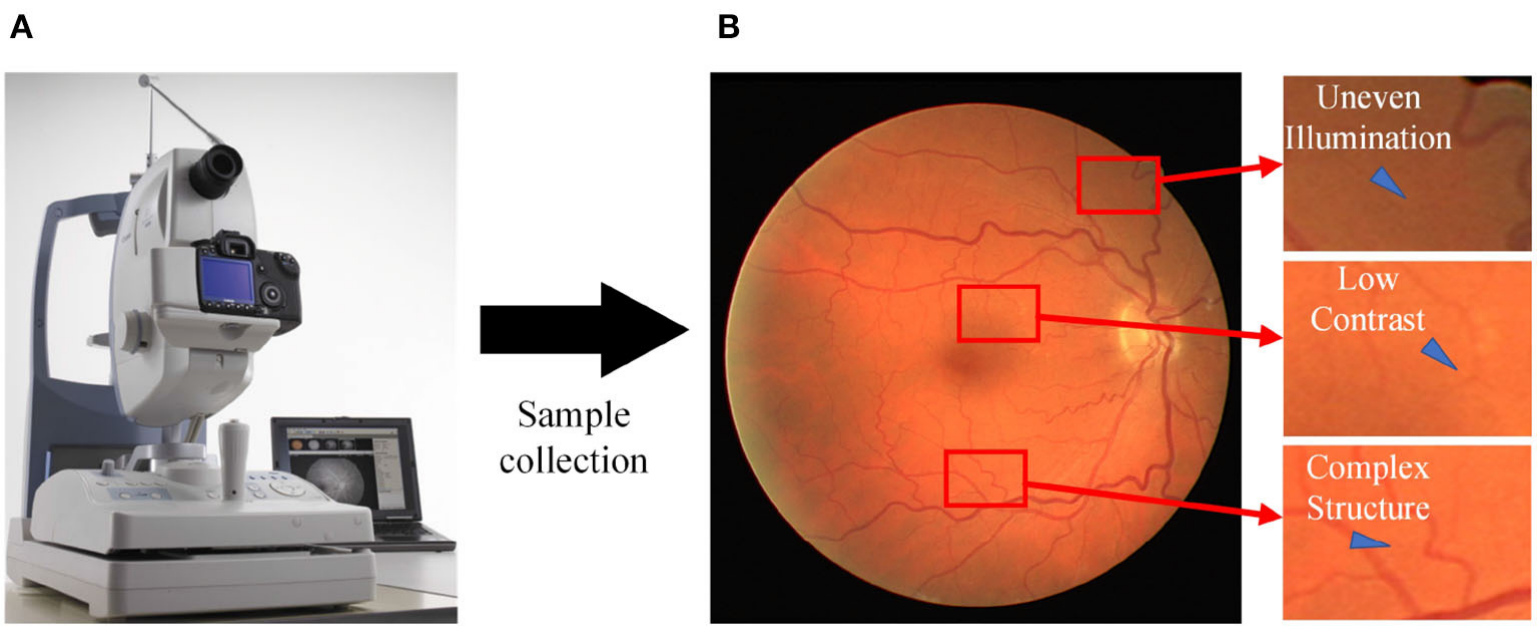
Sampling process for fundus images. **(A)** A fundus camera; **(B)** an acquired sample image with various issues, including uneven illumination, low contrast, and the presence of complex vessel structures.

Fundus diseases may cause vision loss, visual field defect, and serious cases may lead to blindness. Common fundus diseases, such as macular degeneration, hypertensive retinopathy, and diabetic retinopathy (Lin et al., 2021; Badawi et al., 2022; Saranya et al., 2022), are characterized by morphological changes in the retinal vasculature during advanced stages, including optic disc atrophy, vascular proliferation, and macular hole formation. Currently, medical devices are being updated and replaced gradually. Fundus cameras provide an intuitive and easy way to detect and observe the eyes, enabling timely identification of certain fundus diseases that may not have been clearly characterized. They serve as the primary diagnostic tool for ophthalmologists and are widely utilized. However, the manual segmentation of retinal vessels in fundus images (Figure 1) by ophthalmologists is time-consuming and susceptible to subjective errors due to the low contrast and complex structure of the vessels (Liu et al., 2022a). The development of an automated algorithm for vessel segmentation shows great potential in enhancing the capabilities of fundus cameras, reducing the diagnostic burden on physicians, and improving diagnostic efficiency.

Advancements in artificial intelligence are propelling the progress of medical care (Miotto et al., 2017; Mazlan et al., 2021). This technology offers convenient solutions for medical image analysis and disease diagnosis, benefitting both medical professionals and patients (Monemian and Rabbani, 2021; Chen et al., 2022; Liu et al., 2022b). Researchers can automate vessel segmentation through the development of computer-aided medical systems that utilize deep neural networks (Jiang et al., 2017), such as fully convolutional neural networks (FCN) (Jiang et al., 2019, 2020), U-Net (Gegundez-Arias et al., 2021; Wang et al., 2021; Lin et al., 2022; Pan et al., 2022) and generative adversarial networks (Guo et al., 2020; Kamran et al., 2021).

Although existing methods have achieved excellent segmentation performance and generalization ability, there are still unresolved issues. For example, if vessels with a width of less than three pixels are defined as microvessels and the rest are defined as macrovessels, nearly 70% of the pixels in the image can be classified as macrovessels (Yan et al., 2019). Such class imbalance makes existing methods unable to segment complete microvascular structures or even ignore the existence of microvessels, while microvessel segmentation plays a crucial role in the diagnosis of retinal diseases related to vascular proliferation. Moreover, due to the limited availability of publicly available data, deep neural networks may tend to overfit the training data, resulting in poorer generalization ability (Su et al., 2022).

In this work, we propose a retinal vessel segmentation network (MFA-UNet) based on multi-scale feature fusion and attention mechanism. The primary goal is to improve the segmentation performance of vessels under low contrast and preserve the complete microvascular structure. To alleviate the performance bottleneck caused by data scarcity, an image patch-based training method is applied for data augmentation to expand the training samples. MFA-UNet uses UNet as the backbone network and proposes three modules to improve the accuracy and sensitivity of vessel segmentation. First, the multi-scale fusion self-attention module (MSAM) is used to fuse multi-scale features in the skip path and build long-range dependency relationships of the fused features through a self-attention mechanism targeting spatial and channel dimensions, thus improving the ability to preserve complete vascular structures. In addition, different branches in the multi-scale decoding module achieve segmentation of blood vessels with different widths through deep supervision, and integrate and refine these different segmentation results. Finally, considering that the features in the skip paths may cause information redundancy in the decoder, we introduce a Parallel Attention Mechanism (PAM) to filter redundant information in the decoder. The combination of these three modules improves the generalization ability of MFA-UNet and its ability to capture fine microvascular features.

**Figure 2:**
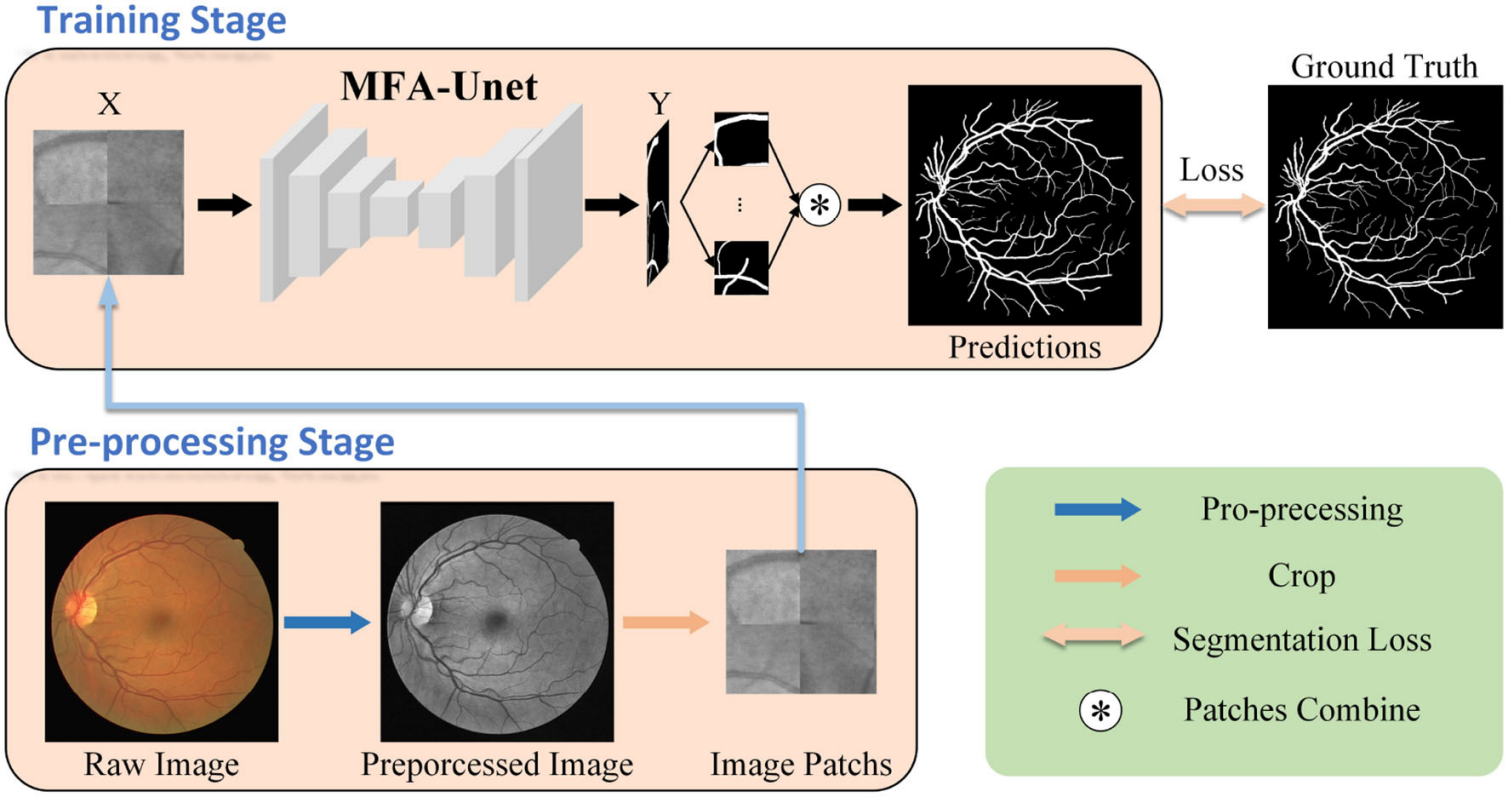
Workflow of the proposed retinal vessel segmentation method. Initially, the fundus image undergoes preprocessing and is divided into multiple image patches, which serve as inputs to MFA-UNet. The network then generates segmentation masks and computes the segmentation loss by comparing them with the ground truth, facilitating network optimization.

The workflow of the proposed retinal vessel segmentation system is illustrated in Figure 2. First, preprocessing techniques are used to improve the visibility of the vessel structure in the images. Subsequently, the training data volume is increased by dividing the image into patches through cropping. These cropped patches are used to train the model and generate the segmentation mask. Finally, the segmented masks are merged to reconstruct the complete retinal vessel segmentation map. The contributions of this paper are summarized as follows:

- In order to address the difficulty of retinal vessel segmentation,we propose a U-Net network structure based on a multi-branch decoder module (MBDM) and a multi-scale feature fusion self-attention module (MSAM) to enhance the effectiveness of retinal vessel segmentation.
- We design a novel channel self-attention mechanism and combine it with a spatial self-attention mechanism to adapt multi-scale features and efficiently learn the dependencies of channel and spatial dimensions.
- We employ a deep-supervised multi-branch decoding module to separate the segmentation of macrovessels and microvessels, thereby achieving precise microvessel segmentation.
- We employ image patching to expand the training dataset, thereby mitigating the issue of overfitting resulting from limited data volume.
- We thoroughly evaluate the proposed model on six publicly available datasets (DRIVE, CHASEDB1, STARE, HRF, IOSTAR, and FIVES) and compare it with other state-of-the-art methods to demonstrate its robustness and effectiveness.

The rest of the paper is structured as follows: Section 2 provides an overview of related work. Section 3 outlines the structure of the proposed MA-UNet model and the techniques used. Section 4 presents the experimental results, demonstrating the effectiveness of the proposed PAM, MASM, and MBDM models. Section 5 provides a discussion and analysis of the experimental results. Finally, in section 6, we draw our conclusions.

## 2 Related works

Retinal vessel segmentation algorithms include unsupervised and supervised methods based on the learning approach, but these methods often fail to achieve high accuracy in microvessel segmentation. Many researchers have incorporated deep supervision techniques and attention mechanisms into supervised methods to improve segmentation results. This section provides a comprehensive review of the relevant literature, covering four aspects: (1) unsupervised methods, (2) supervised methods, (3) attention mechanisms, and (4) deep supervision techniques.

### 2.1 Unsupervised methods

The automatic retinal vessel segmentation algorithm comprises both supervised and unsupervised learning algorithms (Yan et al., 2018). Unsupervised methods utilize inherent vessel characteristics, such as curvature, width, color, etc., as supplementary information for vessel segmentation. Kande et al. (2010) proposed a threshold-based method for retinal vessel segmentation. They utilized spatially weighted fuzzy C-mean clustering and enhanced intensity using data from the green and red channels of the image. Additionally, they improved the contrast between vessels and the background through matched filtering. Zhang et al. (2016) proposed a retinal vessel segmentation method based on the locally adaptive derivative filter, which enhances the segmentation accuracy of macrovessels. Garg et al. (2007) performed vessel segmentation using the global maximum inter-class variance threshold and the ISODATA local threshold. They also improved image contrast and vessel features through pre-processing methods, including adaptive histogram averaging, filtering, and the Hessian matrix. Mahapatra et al. (2022) conducted image enhancement using the Frangi vascular function, achieving superior segmentation results by employing an adaptive weighted spatial fuzzy c-means clustering technique for vessel segmentation. Although these methods are straightforward, efficient, and easily interpretable, they still exhibit limitations in segmentation accuracy.

### 2.2 Supervised methods

Supervised learning methods, including support vector machines, Gaussian models, logistic regression, and artificial neural networks, utilize manually labeled segmentation masks as the optimization target, leading to superior accuracy compared to traditional methods (Li et al., 2016). Ricci and Perfetti (2007) employed image vectorization based on pixel grayscale values to train a Support Vector Machine, resulting in improved accuracy in automatic vessel segmentation. Palanivel et al. (2020) introduced a retinal segmentation method that utilizes the Holder index and Gaussian mixture model classifier. Initially, the Holder index was employed to quantify vessel features, followed by vessel segmentation using the Gaussian mixture model. These methods heavily rely on manually extracted features to achieve exceptional performance but often exhibit insensitivity toward microvessels.

Deep learning-based methods have demonstrated superior accuracy in vessel segmentation compared to machine learning approaches. For instance, Skip FCN (Liu et al., 2019) enhances the segmentation accuracy of microvessels by incorporating skip connections. Samuel and Veeramalai (2021) introduced a vessel segmentation method based on VGG16 Simonyan and Zisserman (2014) utilizing the VSC module to extract and transfer features from the region of interest (ROI). However, the continuous down-sampling operations employed in these methods can lead to the loss of image details and pose a bottleneck to segmentation accuracy. Kar et al. (2023) employed an adversarial generative network for vessel segmentation. The generator synthesizes the vessel mask, while the discriminator distinguishes between the synthesized and real masks, thereby optimizing the segmentation performance of the generator.

Moreover, some researchers have focused on the utilization of multiscale features in the segmentation process to recover the vessel structure. For example, CcNet Feng et al. (2020) had introduced cross-connections to allow the decoder for learning with multi-scale features in the decoding process, thus improving the accuracy of microvessel segmentation. Zhao et al. (2021) proposed a multi-scale upsampling attention module and incorporated it into the U-Net model for cross-scale information transfer. Guo et al. (2019) had designed a multi-scale deep supervision network BRS-DSN with a short connection and enabled to utilize of multi-scale features in the output layer. Deng and Ye (2022) substituted the convolution kernel in the UNet structure with deformable convolution, constructed several skip links, and introduced a channel attention mechanism to extract the multi-angle feature information of the image. Inspired by these works, we proposed a module (MSAM) that integrates multi-scale features and constructs the global correlation.

### 2.3 Attention mechanism

The neural network takes in an entire image as input and learns semantic information through the convolutional kernel, which consists of both relevant and redundant features (Pang et al., 2021). However, redundant information can negatively impact the performance of network in the given task. To address this, the attention mechanism is introduced to selectively emphasize crucial areas in images and filter out redundant information. The attention mechanism can be categorized into local attention and non-local attention. Local attention comprises the channel attention mechanism, spatial attention mechanism, and other mechanisms that facilitate feature selection based on local information. For instance, Guo et al. (2020) introduced the SA-Unet model, which incorporates a spatial attention mechanism to enhance the preservation of microvessel structures and achieve higher accuracy. Non-local attention generally refers to an attention mechanism that captures dependencies between distant features through global information statistics, with the self-attention mechanism being particularly notable. The self-attention mechanism explores correlations within an image to effectively preserve the structure of vessel branches and the vessel tree. Several studies have demonstrated this, including Pang et al. (2021), who employed the self-attention mechanism to adjust multi-scale features and facilitate the restoration of microvessel structures during the decoding process. Similarly, Shen et al. (2022) substituted the convolutional layer with the self-attention mechanism in UNet, leading to outstanding segmentation results and enhanced feature extraction efficiency. Liu et al. (2023)introduced an attentional fusion block to enhance a skip connection, thus improving multiscale feature representation and enabling precise retinal vessel detection. Ouyang et al. (2023) addressed the issue of losing fine-vessel features by employing a local feature enhancement module and attention block to expand the receptive field and optimize the capturing of microvessel features.

In this paper, we introduce the self-attention mechanism to construct correlation between vessel branches and vessel trees. Considering the correlation between channel dimensions, which is ignored in the above methods, we propose a channel self-attention mechanism to capture non-local information of channel dimensions. Additionally, we introduce a parallel attention mechanism into the decoder to effectively utilize the features from the skip path.

### 2.4 Deep supervision

Deep supervision (DS) technology (Lee et al., 2014; Szegedy et al., 2015) was developed to address issues such as gradient vanishing and slow convergence in neural network training. Its fundamental concept involves incorporating auxiliary classifiers into intermediate layers of the model to leverage shallow features in downstream tasks and facilitate the seamless flow of gradients into the deep layers of the network (Zhou et al., 2022). The model generated by the auxiliary classifier can be considered a sub-model of the original model. Through the introduction of additional loss functions, each sub-model can be optimized and specialized for different tasks, thereby enhancing their performance and reducing bias in deep features related to the task. DS techniques have also been utilized in methods presented by Mo and Zhang (2017), He et al. (2021), and Cao et al. (2023) to incorporate multi-scale information and improve the performance and robustness of models in target region segmentation tasks. However, the current application of DS techniques primarily focuses on low-resolution boundary segmentation of target regions, which can be influenced by features in the skip path.

Inspired by these ideas, in order to improve the segmentation accuracy of MFA-UNet on tiny vessels, we introduce MBDM at the end of the decoder. This is done to prevent interference from other features and perform three sub-tasks: macrovessel segmentation, microvessel segmentation, and vessel structure segmentation. We gradually improve the complexity of sub-tasks and integrate sub-targets with different focuses to ensure the integrity of vessel structure in segmentation results.

## 3 Methodology

### 3.1 Overview

The proposed model utilizes U-Net as the backbone model. Due to the limited number of pixels in microvessels, the segmentation of vessel structure becomes inefficient. Additionally, the raw semantic information in the images may not be readily available in the deeper layers of the model. Therefore, a specialized model design is required for the vessel segmentation task.

**Figure 3:**
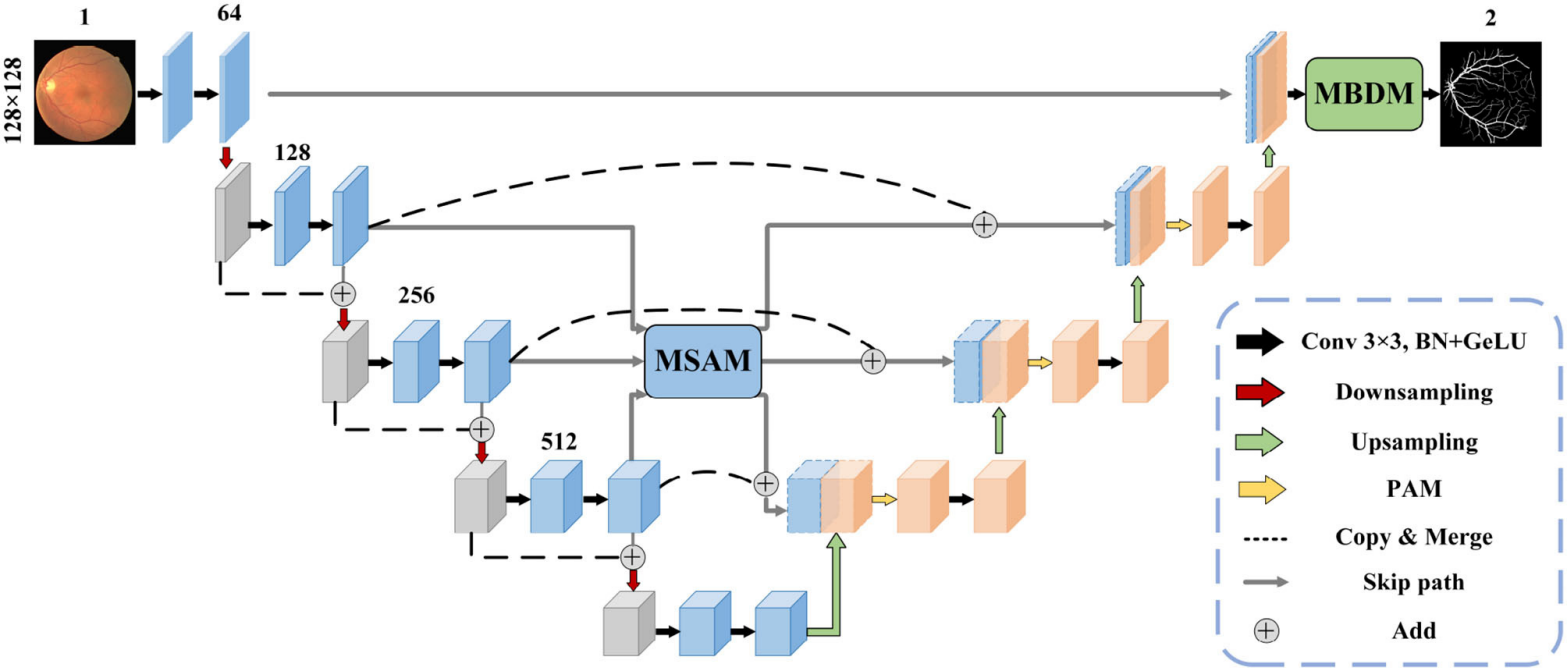
The proposed MFA-UNet model architecture.

The structure of the MFA-UNet is depicted in Figure 3. The network consists of an encoder and a decoder, where the encoder learns from input image patches. The features at different scales in the encoder are fused and sent to MSAM via skip paths. MSAM applies a self-attention mechanism to establish long-range dependencies and generates a weight matrix to adjust the importance of pixels and channels in the fusion features. These fusion features are then forwarded to the decoder for detailed reconstruction of the segmentation mask. During this process, a parallel attention mechanism is applied to filter out redundant features. Finally, MBDM uses different branches to generate macrovessels and microvessels masks from the output features of the decoder, while the fusion branch synthesizes the macrovessel and microvessel structures to obtain the final segmentation mask. It is important to note that in MBDM, all branches are supervised by the same ground truth.

### 3.2 Multi-scale fusion self-attention module

**Figure 4:**
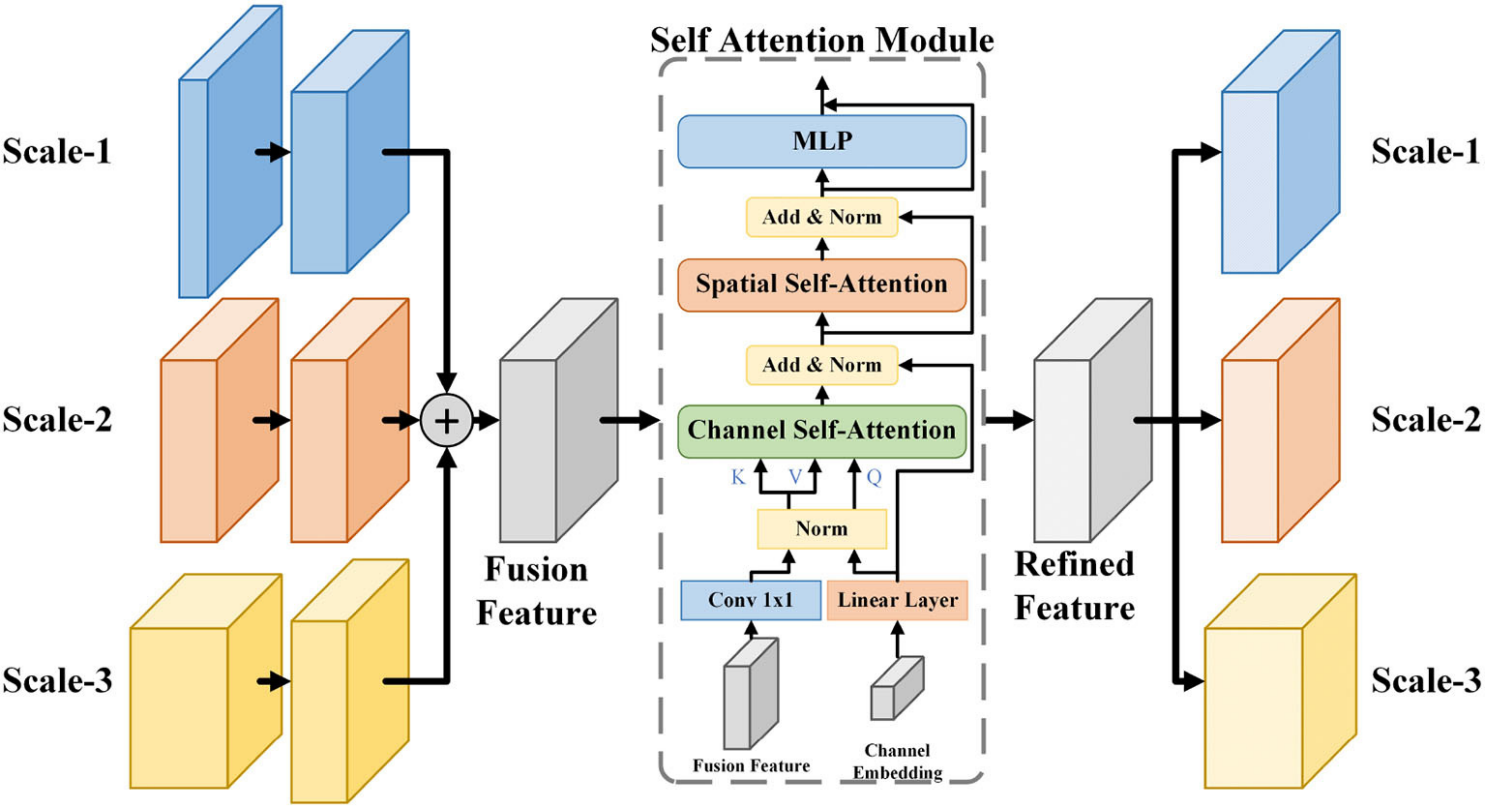
The architecture of MSAM module. It samples and sums the three scales of features to obtain the fusion features, followed by CSA and SSA processing to construct the long-range dependencies of the channel and spatial dimensions, finally integrates the features by using MLP to obtain the refined features, and recovers it to the three scales of the input features by upsampling or downsampling and transfer them to the skip path.

In the microvessel segmentation task, it is common to use features of different scales to restore the edges of the target area and mitigate the information loss caused by downsampling. However, direct fusion of multi-scale features without any adjustment can introduce redundant feature information and affect segmentation performance. To effectively utilize multi-scale information, we propose a multi-scale feature fusion self-attention module (MSAM), which consists of three components: Channel Self-Attention (CSA), Spatial Self-Attention (SSA), and Multi-Layer Perceptron (MLP). The structure of the MSAM is illustrated in Figure 4.

Firstly, the three feature maps, denoted as *F*_1_, *F*_2_, *F*_3_, are reshaped to the same size as *F*_2_ through upsampling or downsampling. Since feature maps with a 64-channel structure contain less semantic information, only feature maps with 128, 256, and 512 channels are then considered for subsequent operations. Subsequently, these feature maps are combined to yield the fusion feature *F*_*a*_. The fusion feature *F*_*a*_ is encoded into *key* and *value* through a 1 × 1 convolution and linear layer. Furthermore, we establish a set of trainable channel embeddings represented by *ch*, which are encoded and transformed into queries using linear layer. In the CSA layer, the correlations between the channel dimensions of the fusion feature *F*_*a*_ are computed using the generated *query* and *key*. This process activates the channel weight map *M*_*c*_ through a softmax operation. *Value* are then element-wise multiplied with *M*_*c*_ to obtain the adjusted feature map *F*_*ch*_. The operations within the CSA layer are described by Equation 1.


(1) $$\begin{array}{l} K=zW_{K},V=zW_{V},Q=chW_{Q},\\ where\;W_{k},W_{B},W_{Q}\in R^{D\times D}\\ F_{ch}=CA(Q,K,V)=ch+softmax(\frac{QK^{T}}{\sqrt{D}})V \end{array}$$

Here, *W*_*K*_ and *W*_*V*_ denote the weights of the linear layers used to encode the token feature *z*, while *W*_*Q*_ represents the weights of the linear layer used to encode the channel embeddings, *R*^*D*×*D*^ denoted a matrix of size *D*×*D*.

The SSA layer consists of a self-attention mechanism that performs linear operations by dot product to mine the information in the image and generate a weight matrix of spatial dimensions, adjusting the importance of each pixel. Finally, the MLP layers enable the transfer of feature information across channels by using combinations of information in channel dimensions. Self-attention mechanisms can establish dependencies between the vascular tree and the pixels of the vascular branches. MSAM integrates feature information at different scales and applies self-attention mechanisms in the spatial and channel dimensions of feature maps to establish long-range correlations between pixels, thereby improving the effect of vessel segmentation. The specific operations performed in MSAM are shown in the Algorithm 1.

**Algorithm 1:** Self-attention based multi-scale feature fusion algorithm.

**Input:** Feature map while down-sampling *F*_*i*_ (i∈[1, 2, 3]), the channel embedding *ch*
**Output:** Multi-scale fusion features *S*_*i*_ (i∈[1, 2, 3])
1: Sample *F*_1_ and *F*_3_ to become the same scale as *F*_2_;
2: Apply a 3 × 3 convolution on *F*_1_ and *F*_3_ to change the number of channels to 256;
3: Obtain fusion feature *F*_*a*_ by perform the sum $\overset{3}{\underset{i=1}{\sum }}F_{i}$;
4: Apply 1 × 1 convolution to reshape *F*_*a*_ into the token feature *z*. Then, perform linear operations on of the token feature *z* by dot product to generate *key* and *values*;
5: Perform a dot product operation on *key* and channel embedding *ch* as *queries* to obtain *W*_*ch*_;
6: Conduct matrix multiplication on *W*_*ch*_ and *values* to obtain *S*_*ch*_;
7: Perform a dot product operation on *S*_*ch*_ to generate v*values*, *key* and *queries*;
8: Perform a dot product operation between *queries* and *key* to derive *W*_*sp*_, and perform a dot product operation between *W*_*sp*_ and *values* to derive *S*_*sp*_;
9: Impose linear calculations on *S*_*sp*_ to obtain *S*;
10: Obtain *S*_*i*_ for the sampling operation of *S*, where *S*_*i*_ is reshaped to be the same size as *F*_*i*_ (i∈[1, 2, 3]);

### 3.3 Multi-branch decoder module

**Figure 5:**
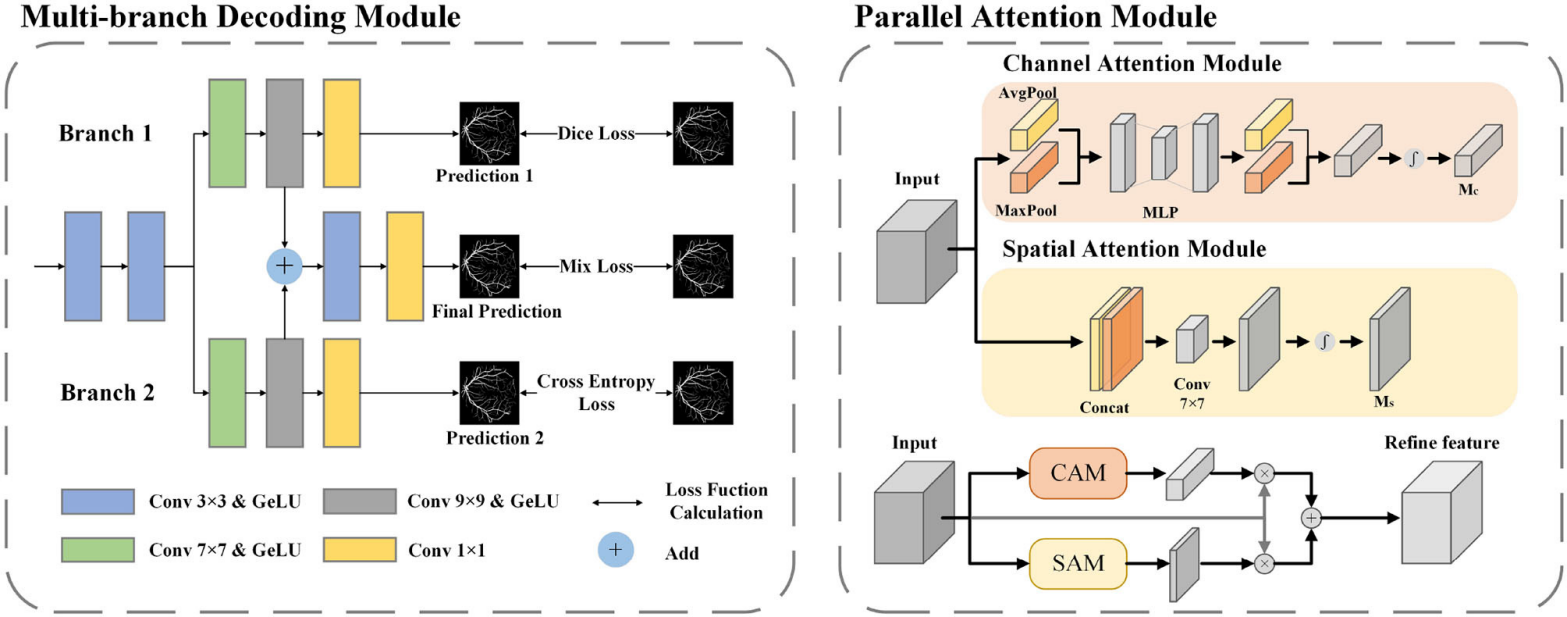
The architecture of MBDM and PAM. In MBDM, Branches 1 and 2 receive the feature maps from MFA-UNet as input and are supervised with different loss functions to achieve the segmentation of both macro- and micro-vessels. The fusion branch combines the outputs of branches 1 and 2 and adjusts them to obtain the final prediction. In PAM, the input features are sent to CAM and SAM to adjust the weights of the channel and spatial dimensions, respectively. This adjustment helps filter out redundant information. The resulting features are then added together to achieve mutual complementation of feature information.

In fundus images, nearly 77% of the vessel pixels belong to macrovessels, while only 23% belong to microvessels. This causes an imbalance of vessel proportions in the images, which makes the single network structure less accurate in the segmentation of microvessels (Yan et al., 2019). To solve this imbalance problem, we applied deep supervision technology to the decoder. By adding multiple branches before the output layer, the vessel segmentation is divided into three stages: macrovessel segmentation, microvessel segmentation, and vessel fusion. We describe this structure as a multi-branch decoder module, and the structure is shown in Figure 5.

Branch 1 is optimized by the Binary Cross Entropy (BCE) function for pixel-level loss. The function compares the predicted map with the ground truth pixel by pixel, and the imbalance in vessel proportion causes the function to penalize macrovessels more, making this branch better for macrovessel segmentation. The optimization of branch 2 is implemented by dice loss (Milletari et al., 2016) for *f* class-level loss. The dice loss maximizes the intersection of the segmentation result and the ground truth as the optimization objective, which reduces the negative impact caused by the imbalance of vessel proportions and makes the branch better for preserving the microvessel structure. In order to recover the broken vascular structure, we aim to gather local information by utilizing a convolutional kernel with a larger receptive field. To achieve this, we introduce convolutional layers of 7 × 7 and 9 × 9 in branches 1 and 2. This allows for a broader scope of information aggregation, aiding in the restoration of the vascular structure. The module merges the outputs of branches 1 and 2 to obtain a segmentation map that considers both macro and microvessel segmentation. The segmentation map is then used as input to the fusion branch, and convolutional layers with kernel sizes of 3 × 3 and 1 × 1 are applied to adjust the segmentation map. The 3 × 3 convolution kernel is used to adjust the fusion features and restore them based on vessel locality, while the 1 × 1 convolution kernel is used to adjust individual pixel values. The fusion branch needs to consider both macro and microvessel segmentation, so a mixed loss function of BCE and Dice loss is used for training. The BCE loss and the Dice loss are calculated as follows:


(2) $$\begin{array}{l} BCE\;Loss=-\frac{1}{N}\sum_{c=1}^{C}\sum_{n=1}^{N}y_{n,c}logp_{n,c}\\ Dice\;loss=1-\frac{1}{N}\sum_{c=1}^{C}\sum_{n=1}^{N}\frac{2\|y_{n,c}p_{n,c}\|+smooth}{\|y_{n,c}^{2}+p_{n,c}^{2}\|+smooth}\\ p_{n,c}\in P,y_{n,c}\in Y \end{array}$$

where *p*_*n, c*_ and *y*_*n, c*_ are the target label and prediction probability of the cth category with the nth pixel in the batch, *Y* is the ground truth, *P* is the prediction result, *C* is the number of categories, and *N* is the total number of pixels in the dataset in the batch.

### 3.4 Parallel attention module

In the structure of U-Net, the input of the convolutional layer of the decoder is the fusion feature after cascading the output of the previous layer with the encoder feature, which contains many useless information such as the optic disc feature. In this paper, a parallel attention module (PAM) is included in the decoder to adjust the spatial and channel dimensions of the feature maps respectively, therefore reducing the redundant information and allowing the network to focus on the segmentation of blood vessels.

In the Channel Attention Mechanism (CAM) (Woo et al., 2018), both a max-pooling and average-pooling operation are performed along the spatial dimension to extract different feature information, and two feature maps with size 1 × 1 are cascaded input to the MLP and sigmoid activation layer to obtain the channel dimension weight matrix. This operation avoids the influence of spatial dimensional information. The Spatial Attention Mechanism (SAM) (Woo et al., 2018) also applies maximum pooling and average pooling to the channel dimensions to avoid the influence of spatial information, to extract different spatial dimensions of information, and cascades the features through a 7 × 7 convolutional layer to obtain the spatial dimension dependency, and finally passes through a sigmoid activation layer to obtain the spatial dimension weight matrix. We parallel the CAM and SAM to complement the feature map with the different dimensions. The architecture of the attention module used in this paper is shown in Figure 5.

The formulas for the channel attention mechanism and the spatial attention mechanism are as follows:


(3) $$\begin{array}{l} M_{c}(F)=\sigma (M(AvgPool(F))+M(MaxPool(F)))\\ \;=\sigma (W_{1}(W_{0}(F_{avg}^{c}))+W_{1}(W_{0}(F_{max}^{c}))) \end{array}$$

(4) $$\begin{array}{l} M_{s}(F)=\sigma (f^{7\times 7}([AvgPool(F);MaxPool(F)]))\\ \;=\sigma (f^{7\times 7}([F_{avg}^{s};F_{max}^{s}])) \end{array}$$

where *M* denotes the operation performed by the MLP, *F* is the input feature map, *W* denotes the weight of the fully connected layer in the MLP, *f*^7 × 7^ is the weight value of the convolution kernel of 7 × 7, and σ as the sigmoid activation function. *M*_*c*_(*F*) is the weight matrix generated by channel attention, and *M*_*s*_(*F*) is the weight matrix generated by spatial attention.

Where *M* represents the operation performed by the MLP, *F* denotes the input feature map, *W* signifies the weight of the fully connected layer in the MLP, *f*^7 × 7^ represents the weight value of the 7 × 7 convolution kernel, and σ denotes the sigmoid activation function. *M*_*c*_(*F*) denotes the weight matrix generated by channel attention, while *M*_*s*_(*F*) represents the weight matrix generated by spatial attention.

## 4 Experiments

### 4.1 Datasets

In this paper, we evaluated the performance of the proposed method, the baseline U-Net, and other segmentation methods based on U-Net variants using six public datasets: DRIVE, STARE, CHASE-DB1, HRF, IOSTAR, and FIVES. We trained and tested MFA-UNet, U-Net, and other methods separately on four different fundus image datasets. The DRIVE dataset consists of 40 fundus images, with 20 images for training and 20 images for testing. The STARE dataset (Hoover et al., 2000) contains 20 fundus images, including some images with lesions. We randomly selected 10 images for training and 10 images for testing. The CHASE-DB1 dataset (Owen et al., 2009) consists of 28 images, 20 of which are used for training and the rest for testing. The HRF dataset (Odstrcilík et al., 2013) contains 15 normal fundus images, 15 fundus images with diabetic retinopathy, and 15 fundus images with glaucoma, of which 35 images were used for training and the remaining images for testing according to Shen et al. (2022). The OSTAR dataset (Zhang et al., 2016) comprises 30 retinal images, with 20 images for training and 10 images for testing. The FIVES dataset (Jin et al., 2022) encompasses retinal images depicting various conditions, including health, diabetic retinopathy, glaucoma, and age-related macular degeneration. Moreover, corresponding vascular segmentation masks are provided for each image. Each category contains a total of 200 images. The dataset is partitioned into 600 images for training and 200 for testing. Each of the six publicly available datasets is described in detail in Table 1.

**Table 1:** Descriptions of the DRIVE, STARE, CHASE-DB1, HRF, IOSTAR, and FIVES datasets.

**Dataset**	**Quatity**	**Resolution**	**Train-test**
DRIVE	40	565 × 584	20–20
SRARE	20	700 × 605	10–10
CHASE-DB1	28	999 × 960	20–8
HRF	45	3, 504 × 2, 336	35–10
IOSTAR	30	1, 024 × 1, 024	20–10
FIVES	800	2, 048 × 2, 048	600–200

### 4.2 Preprocessing

During the imaging process of fundus cameras, various factors such as room lighting and physician expertise can lead to uneven illumination and low contrast in fundus images, which can hinder the segmentation of retinal vessels. Therefore, preprocessing techniques are considered essential to improve the quality of fundus images. First, the NTSC conversion method is used to convert the RGB image to a grayscale image since the green channel contains more significant information than the red and blue channels. The formula for this conversion is as follows:


(5) $$\begin{array}{l} Gray=0.299*R+0.587*G+0.114*B \end{array}$$

**Figure 6:**
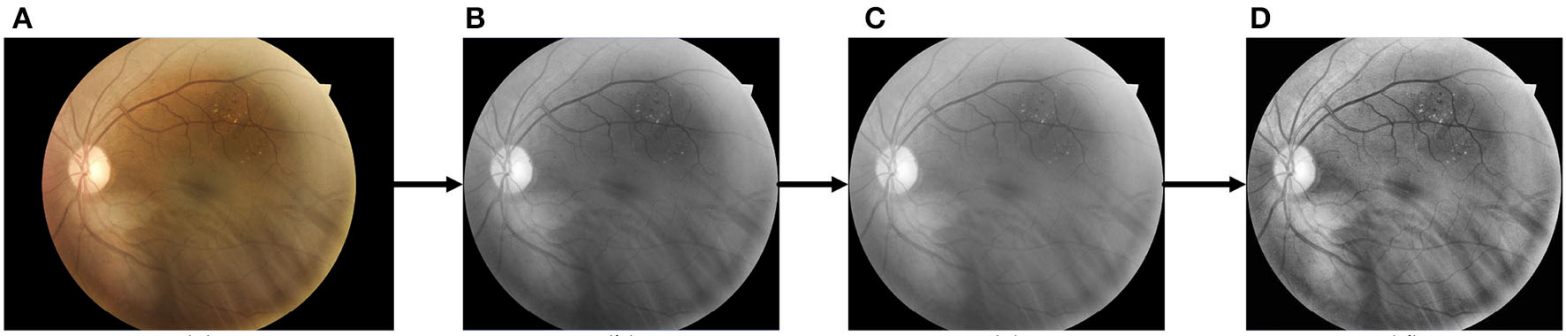
The pre-processing process for retinal images. **(A)** Raw image; **(B)** cropped and grayscaled image; **(C)** Gamma-corrected image; **(D)** CLAHE processed image.

To address the problem of low contrast in the vessels, we apply the Contrast-Limited Adaptive Histogram Equalization (CLAHE) technique to the images. This method effectively enhances the vessel features by equalizing the grayscale histogram of a specific portion of the image. In addition, we use gamma correction to brighten the image with a gamma value of 1.2, making the darker areas of the image more visible. The raw and pre-processed images are shown in Figure 6.

### 4.3 Data augmentation

Due to the high resolution of the images in each dataset, using the entire image as input is likely to place a significant burden on the hardware, and the availability of training data is limited. To solve this problem, we extend the training data using the overlap method. Briefly, we use a window with a size of 128 by 128 and a step size of 15 to perform patch cropping for each image and generate 25,000 patches with an overlap of 87.5% for adjoining image patches. We then used random cropping, horizontal and vertical mirror inversion, and random angle rotation for data enhancement to improve sample diversity.

### 4.4 Implementation details

Our experiment is performed using the PyTorch framework. All models are trained and tested on an NVIDIA RTX 3080 with 24G of memory. The MFA-UNet model is optimized using the Adam optimizer with the parameters β_1_ = 0.9;β_2_ = 0.99. During both the training and testing phases, the batch size is set to 32, and the initial learning rate is set to 5 × 10^−4^. To facilitate the training phase, a learning rate decay strategy is implemented with a decay period of 50 epochs and a decay rate of 0.1. We designate 10% of the training data as the validation set to evaluate the performance of MFA-UNet. The models with the highest the area under the ROC curve (AUC) in the validation set are saved for model performance evaluation.

### 4.5 Evaluation metrics

To binarize the segmentation probability map, we utilize a fixed threshold, where pixel values exceeding this threshold are set to 255, and while those below the threshold are set to 0. In the binarized segmentation maps, pixels that are correctly classified as vessels are denoted as true positive (TP), while the pixels that are incorrectly classified as part of vessels are designated as false positive (FP). Pixels that are accurately identified as non-vessel are marked as true negative (TN), and the pixels that are wrongly assigned as non-vessel are recorded as false negative (FN). To evaluate the proposed model, we employ several widely used metrics, including Accuracy (Acc), Dice Similarity Coefficient (DSC), Sensitivity (Sen), and Specificity (Sp). The formulas for each metric are presented below:


(6) $$\begin{array}{l} Acc=\frac{TP+TN}{N},\;DSC=\frac{2TP}{2TP+FP+FN}\\ Se=\frac{TP}{TP+FN},\;Sp=\frac{TN}{TN+FP} \end{array}$$

where *N* = *TP*+*TN*+*FP*+*FN*. The receiver operating characteristic curve (ROC) is generated by constructing a coordinate system using the true positive rate (Se) and false positive rate (1-Sp). AUC is then calculated to evaluate the overall classification ability of the model.

## 5 Results

In this section, we perform a comparative analysis of the proposed model with other vessel segmentation methods, including U-Net, MS-NFN, CcNet, and NestU-Net, etc. to demonstrate the superiority of our proposed method over other existing techniques. The evaluation results of the proposed model and other methods on the DRIVE, STARE, CHASE DB1, and HRF datasets are presented in Table 2, where mSen and mSp represent the mean values of the sensitivity and specificity of the model on the binary classification task. It is important to note that in each experiment, MFA-UNet and U-Net are trained and tested separately on four different fundus image datasets, while the results of other methods are obtained from the cited references.

**Table 2:** Comparison of the proposed method with other methods in terms of evaluation metrics including mSen, mSp, Acc, AUC, and DSC, using the DRIVE, STARE, CHASE-DB1, HRF, IOSTAR, and FIVES datasets (unit:%).

**Dataset**	**Method**	**Years**	**mSen**	**mSp**	**Acc**	**AUC**	**DSC**
DRIVE	Ronneberger et al. (2015)	2016	79.66	97.80	95.49	97.72	81.72
	Wang et al. (2019)	2019	76.48	98.17	95.41	–	80.93
	Feng et al. (2020)	2020	76.25	98.09	95.28	96.78	–
	Wang et al. (2021)	2021	80.60	92.83	95.12	97.48	78.63
	Naveed et al. (2021)	2021	**81.41**	97.02	95.40	–	–
	Liu et al. (2023)	2023	79.85	97.91	95.61	–	82.29
	Ouyang et al. (2023)	2023	79.83	97.93	95.63	–	82.30
	**Proposed**	2023	81.23	97.81	**95.71**	**98.09**	**82.79**
STARE	Ronneberger et al. (2015)	2016	83.24	97.96	96.38	98.54	83.22
	Wang et al. (2019)	2019	75.30	98.85	96.40	–	81.25
	Feng et al. (2020)	2020	77.09	98.48	96.33	97.00	–
	Wang et al. (2021)	2021	82.30	**99.45**	96.41	96.20	79.47
	Naveed et al. (2021)	2021	82.88	96.53	95.41	–	–
	Deng and Ye (2022)	2022	82.72	98.47	**96.43**	–	–
	Mahapatra et al. (2022)	2022	68.46	98.02	96.01	–	–
	Liu et al. (2023)	2023	80.39	98.36	96.35	–	83.15
	**Proposed**	2023	**85.39**	97.69	96.40	**98.77**	**83.51**
CHASE-DB1	Ronneberger et al. (2015)	2016	77.21	98.82	96.61	98.75	83.42
	Wu et al. (2018)	2018	75.44	98.47	96.37	98.25	-
	Wang et al. (2019)	2019	77.30	97.92	96.03	–	75.91
	Lu et al. (2020)	2020	**81.35**	97.62	96.17	97.82	–
	Naveed et al. (2021)	2021	81.53	97.11	95.61	–	–
	Liu et al. (2023)	2023	80.20	97.94	96.70	–	82.36
	**Proposed**	2023	79.32	**98.86**	**96.71**	**98.86**	**84.17**
HRF	Ronneberger et al. (2015)	2016	76.79	98.30	96.61	98.21	79.46
	Jin et al. (2019)	2019	74.67	98.74	96.51	98.31	–
	Wang et al. (2020)	2020	79.67	95.40	94.88	97.85	–
	Shen et al. (2022)	2021	**83.13**	98.03	96.80	**98.62**	**80.95**
	Mahapatra et al. (2022)	2022	71.12	98.43	96.58	–	–
	**Proposed**	2023	77.33	**98.45**	**96.82**	98.31	78.60
IOSTAR	Ronneberger et al. (2015)	2016	75.79	97.62	95.78	97.27	80.67
	Zhang et al. (2016)	2016	75.45	97.40	95.14	96.15	–
	Kar et al. (2023)	2023	78.07	97.88	96.10	97.67	–
	**Proposed**	2023	**80.33**	**98.15**	**96.32**	**98.21**	**81.75**
FIVES	Ronneberger et al. (2015)	2016	78.77	98.34	96.28	97.58	81.74
	Yan et al. (2018)	2018	77.30	98.73	96.70	97.27	81.65
	**Proposed**	2023	**80.07**	**99.09**	**97.10**	**97.81**	**84.04**

The bolded value is the maximum value in the comparison.

Among the employed evaluation metirics, mSen and DSC represent the degree of overlap between the segmentation prediction of the model and the ground truth label. Accuracy and AUC, on the other hand, serve to evaluate the overall performance of the model in pixel-level classification. Additionally, mSp indicates the segmentation accuracy of the model for non-vessel areas. The completeness of the vascular structure in the segmentation mask is crucial in clinical analysis. Therefore, in the performance comparison, we focus on the superiority and inferiority of MFA-UNet and other methods in the three comprehensive metrics of accuracy, DSC, and AUC.

The comparative results on the DRIVE dataset showcase the superior performance of MFA-UNet over other state-of-the-art algorithms in terms of accuracy, DSC, and AUC, with improvements observed across all metrics when compared to UNet. The DRIVE dataset, with its larger number of test samples, serves as a more representative benchmark for evaluating segmentation algorithms in real-world scenarios. It is worth noting that the method proposed in Naveed et al. (2021) demonstrates higher sensitivity compared to our approach, possibly due to the post-processing effect of the block matching 3D (BM3D) speckle filter, which specifically enhances sensitivity in microvessels. On the other hand, the method proposed by Barkana et al. (2017) exhibits excellent specificity. However, this approach combines predictions from multiple algorithms for vessel segmentation, resulting in the introduction of excessive model parameters and increased inference time.

In the STARE dataset, Nest U-Net, as proposed in Wang et al. (2021), outperforms MFA-UNet in terms of specificity and segmentation accuracy. We believe that Nest U-Net achieves this by utilizing high-resolution feature maps to produce more refined vessel segmentation results. On the other hand, the proposed MFA-UNet achieves higher sensitivity, AUC, and DSC than other models, indicating that MFA-UNet maintains the segmentation effect for macriovessels while preventing the loss of microvessel structures due to the MSAM.

In the CHASE-DB1 dataset, MFA-UNet demonstrates superior performance in all metrics, except for sensitivity, when compared to other current methods. The approach proposed in Lu et al. (2020) outperforms other methods specifically in terms of sensitivity. This method employs two models to separately segment macrovessels and microvessels, and combines the resulting segmentation maps to obtain the final results, allowing for a combined segmentation effect on both macrovessels and microvessels. In contrast, MFA-UNet utilizes the Multi-Branch and Dense Module (MBDM) to target different vessel segmentations and combine the results from multiple branches, resulting in more efficient memory usage compared to an ensemble model. Similarly, the method presented in Shen et al. (2022) surpasses other methods in sensitivity, AUC, and DSC on the HRF dataset by replacing the convolutional layer with a self-attention mechanism, enabling a global perceptual field. However, it should be noted that this method requires significantly higher memory resources compared to other approaches.

In the IOSTAR dataset, our model achieved the highest level of performance in the comparative analysis. The dataset exhibits relatively prominent blood vessels, and the MFA-UNet model successfully segments the vascular structures comprehensively, outperforming the UNet model by effectively restoring interrupted vessels.

In the FIVES dataset, our proposed model has also achieved optimal performance across all metrics. The FIVES dataset comprises images featuring various categories of fundus diseases, where the presence of biomarkers like leptomeningeal fundus, exudation, and hemorrhage can significantly influence vascular segmentation performance. As a result, the sensitivity of MFA-UNet only reaches 80.07%. However, it still excels in terms of DSC and AUC, highlighting the remarkable robustness of MFA-UNet when confronted with the diverse fundus images.

We observed a common issue among all segmentation models evaluated on this dataset: square-shaped convolutional kernels struggle to preserve the curvatures of blood vessels, particularly those with smaller curvatures. We believe that during the encoding process, the model's perception of blood vessels with larger curvatures is compromised due to their relatively low proportion in the overall structure. Additionally, the local feature extraction capabilities of the convolutional kernels fail to accurately segment distributed and highly curved vessel structures.

**Figure 7:**
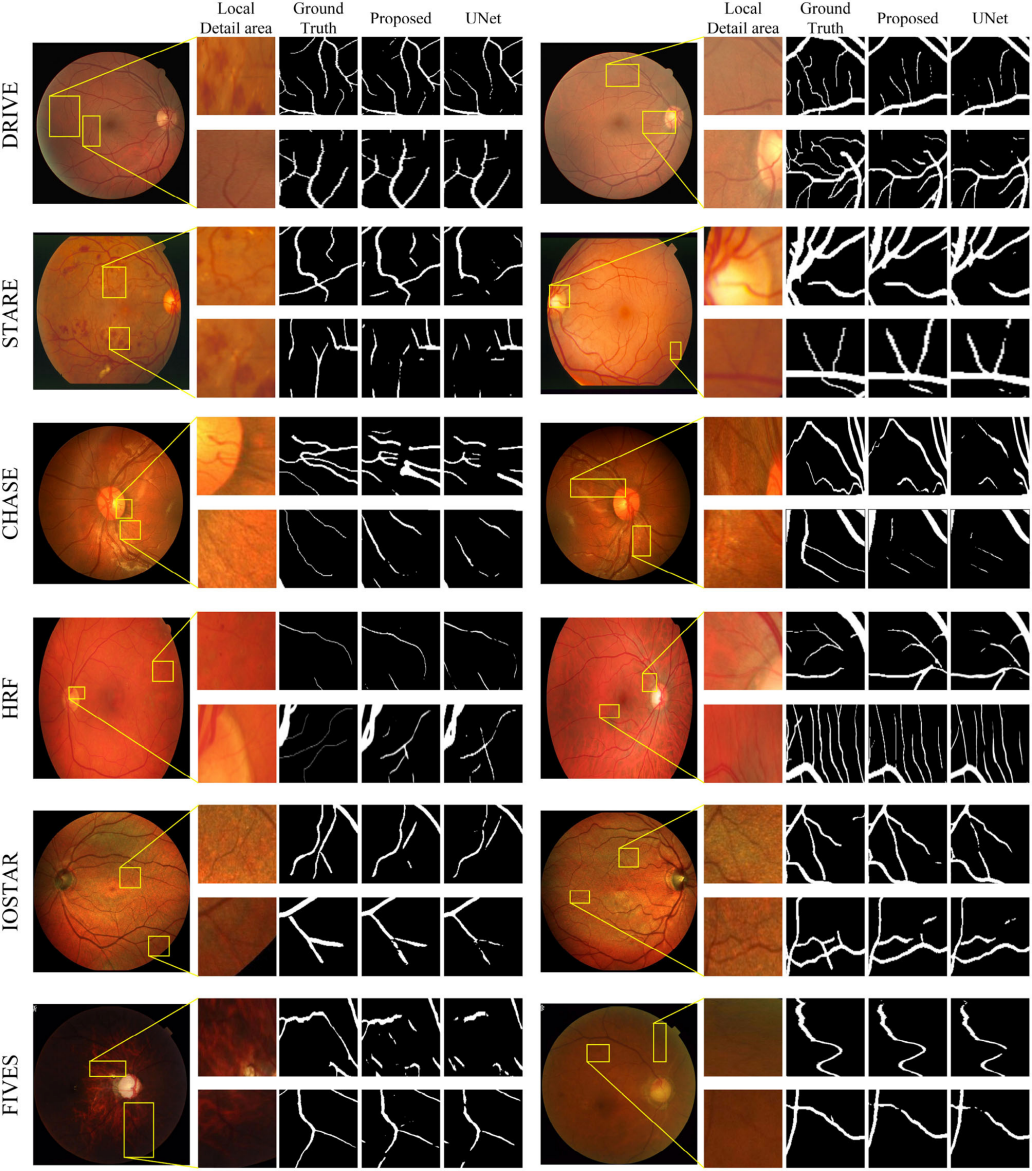
Retinal vessel segmentation results on DRIVE, STARE, Chase-DB1, HRF, IOSTAR, and FIVES datasets. The left and right columns present the segmentation results of different samples from the same dataset. In each column, from left to right, you will find the raw image, its local detail images, ground truth, result of MFA-UNet, and result of UNet, respectively.

Figure 7 displays the segmentation visualization results of MFA-UNet on various datasets. The visual analysis demonstrates the exceptional performance of our method in accurately segmenting microvessel while maintaining their overall structural integrity. Notably, MFA-UNet exhibits precise delineation of macrovessels, highlighting its robust segmentation capability for such structures. To emphasize challenging segmentation regions, such as areas with significant width variations and low contrast in blood vessels, we have zoomed in and positioned these vessels adjacent to each image.

**Figure 8:**
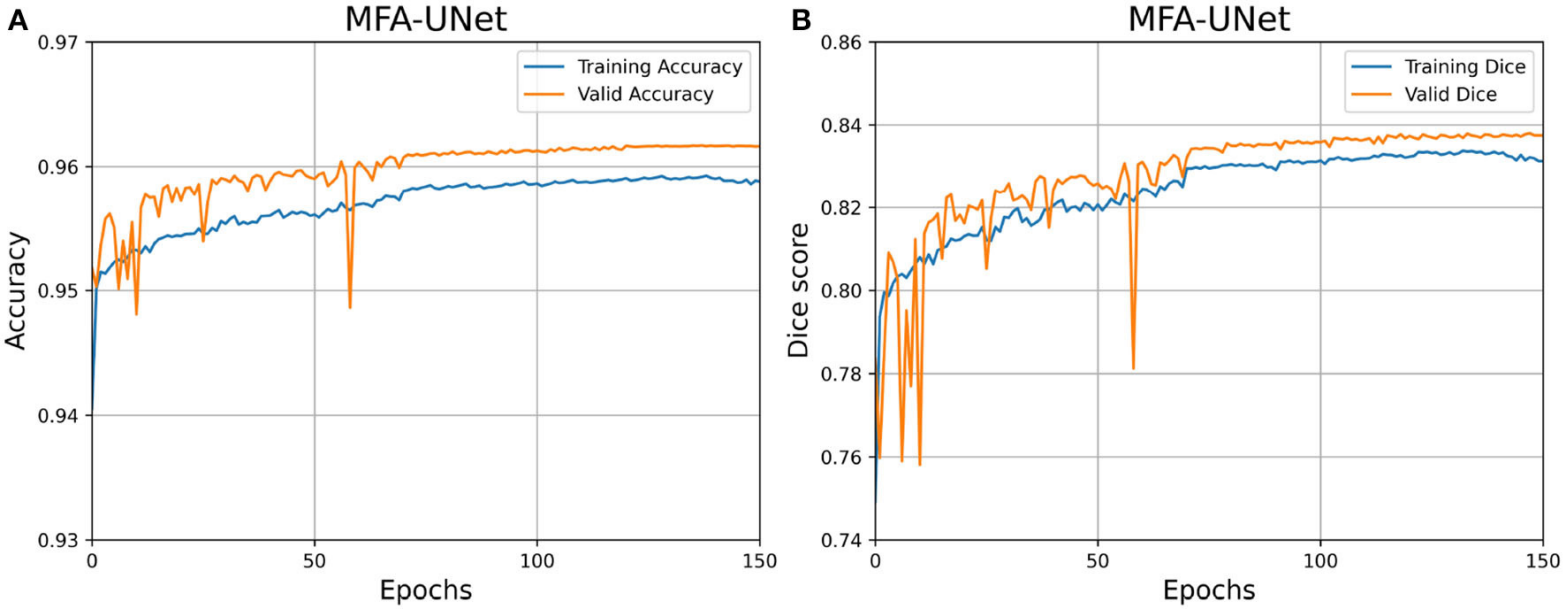
Learning curves for MFA-UNet trained on the DRIVE dataset. **(A)** Learning curve illustrating the changes in Accuracy; **(B)** learning curve illustrating the changes in dice score.

We have depicted the learning curve of MFA-UNet on the DRIVE dataset in Figure 8, aiming to observe the performance variations of MFA-UNet throughout the training process. The learning curve tends to plateau after the 80th epoch, indicating the gradual convergence of MFA-UNet on the training set. Moreover, MFA-UNet did not exhibit significant overfitting during the training process, as evidenced by the similarity between the learning curves of the validation set and the training set. This observation validates the effectiveness of patch-based data augmentation techniques.

### 5.1 Ablative studies

Apart from conducting comparisons with aforementioned methods, we have conducted two sets of ablative experiments to analyze the influence of different combinations of modules and loss functions on the segmentation performance of MFA-UNet. These ablative experiments were exclusively carried out on the DRIVE dataset due to computational resource limitations.

#### 5.1.1 Effect of various module combinations on segmentation performance

In the proposed MFA-UNet, we have incorporated PAM, MSAM, and MBDM to enhance the segmentation performance of the model. To validate the effectiveness of these modules, we conducted ablation experiments. The model that excludes all of the aforementioned modules is referred to as the basebone.

**Figure 9:**
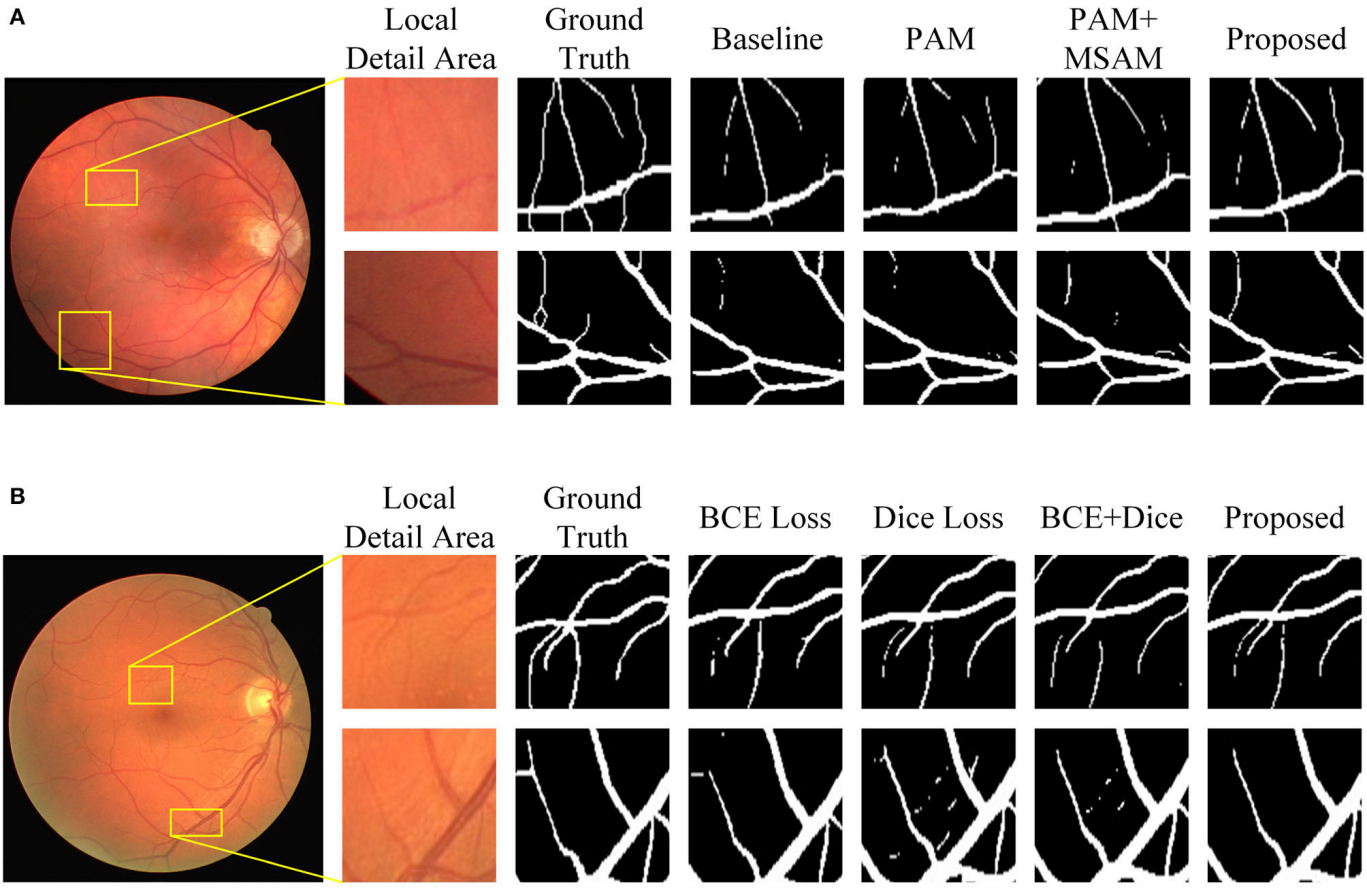
Visualization of segmentation results of MFA-UNet and its ablated versions on the DRIVE dataset. **(A)** Results of various combination of modules, **(B)** results of different loss functions.

Table 3 presents the performance of the models with the addition of each module to the basebone. The backbone model, which incorporates residual connections, large kernel convolution downsampling layers, and adjustments to the number of channels, reduces the number of parameters by 42.7% compared to U-Net while maintaining similar performance. The introduction of PAM into the backbone led to improvements in the sensitivity, accuracy, and AUC of the model. This enhancement can be attributed to improved feature extraction. The incorporation of MSAM significantly improved the sensitivity, which represents the accuracy of the model in blood vessel segmentation. This improvement indicates that multi-scale features can greatly enhance segmentation performance. However, accuracy does not improve, suggesting that the model misclassifies background pixels as blood vessels during pixel class discrimination. Finally, the inclusion of MBDM in the model results in MFA-UNet achieving the best performance in accuracy, AUC, and DSC. In the ablation experiments, we observed significant improvements in certain metrics with the addition of each module to the model. This suggests that incremental enhancements in the model contribute to the improvement of vessel segmentation performance (see Figure 9). By incorporating MBDM into the model, it achieves the highest values in comprehensive metrics and attains the best balance across each metric.

**Table 3:** The quantitative results of the ablative studies.

**Ablation study**	**Method**	**Parameters**	**mSen**	**mSp**	**Acc**	**AUC**	**DSC**
Study 1	U-Net	3.45M	79.66	97.80	95.49	97.72	81.72
	baseline	1.92M	77.94	**98.01**	95.46	97.70	81.38
	+PAM	2.08M	80.61	97.78	95.60	97.88	82.34
	+MSAM	2.34M	**81.93**	97.61	95.61	97.99	82.63
	+MBDM	2.42M	81.23	97.81	**95.71**	**98.09**	**82.79**
Study 2	BCE Loss	2.34M	81.26	97.76	95.66	98.02	82.67
	Dice Loss	2.34M	**83.93**	97.10	95.42	92.86	82.38
	BCE Loss + Dice Loss	2.34M	83.34	97.43	95.64	98.07	82.66
	Proposed	2.42M	81.23	**97.81**	**95.71**	**98.09**	**82.79**

Study 1: Effects of different module combinations on segmentation performance; Study 2: Comparison of segmentation performance for MFA-UNet with MBDM using different loss functions and MFA-UNet without MBDM. The bolded value is the maximum value in the comparison.

#### 5.1.2 Effect of the choice of loss function on segmentation performance

In this set of experiments, we removed the MBDM component from MFA-UNet and trained the network with three different loss functions to verify the effectiveness of MBDM in improving segmentation performance. The visual segmentation results of these networks are shown in Figure 9. When employing the BCE loss function, the network can only segment the structure of coarse vessels well, but causes structural discontinuity in fine vessel segmentation. By employing the Dice loss function, the network achieves a sensitivity of 83.93% and exhibits improved segmentation of small vessels, albeit with some non-vessel areas being erroneously segmented. We noticed that when using a mixed loss function, the misclassification rate in some uncertain areas of the network is significantly reduced, which is manifested as an increase in specificity. Benefiting from the training method of MBDM, MFA-UNet can better preserve the structure of fine vessels and reduce the misclassification rate of non-vessel areas, achieving the highest accuracy, specificity, and DSC in Table 3.

### 5.2 Cross-training experiments

To validate the generalization and robustness of the proposed method, we conduct cross-training experiments. Specifically, we evaluated the performance of a model trained and converged on one dataset when applied to another dataset. Unlike the method of retraining the neural network in Li et al. (2016), we utilize the model trained in Section 4.6 for cross-training without multiple training. The outcomes of the cross-training experiments are presented in Table 4. Notably, for the DRIVE dataset, our method achieves superior sensitivity, specificity, and accuracy compared to other methods, albeit with a lower AUC. Conversely, on the STARE dataset, the specificity and accuracy of our results are inferior to other methods. We attribute this situation to the presence of lesion images in the STARE dataset, which facilitate the learning of sufficient features by the model, while the segmentation masks in the DRIVE dataset exhibit greater detail compared to those in the STARE dataset.

**Table 4:** The results of cross-training experiments on the DRIVE and STARE dataset.

**Test dataset**	**Method**	**mSen**	**mSp**	**Acc**	**AUC**
DRIVE (trained on STARE)	Yan et al. (2019)	74.43	98.14	95.09	**97.20**
	Li et al. (2016)	72.73	98.10	94.86	96.77
	Feng et al. (2020)	72.17	98.20	94.86	93.27
	Jin et al. (2019)	70.77	99.14	94.81	95.68
	Proposed	**74.47**	**98.46**	**95.28**	97.03
STARE (trained on DRIVE)	Yan et al. (2019)	73.19	**98.40**	**95.80**	96.78
	Li et al. (2016)	70.27	98.28	95.45	96.71
	Feng et al. (2020)	74.99	97.98	95.63	96.21
	Jin et al. (2019)	70.00	97.59	94.74	97.43
	Proposed	**85.53**	96.56	95.36	**98.07**

The bolded value is the maximum value in the comparison.

### 5.3 Comparison of parameters, flops, and speeds

**Figure 10:**
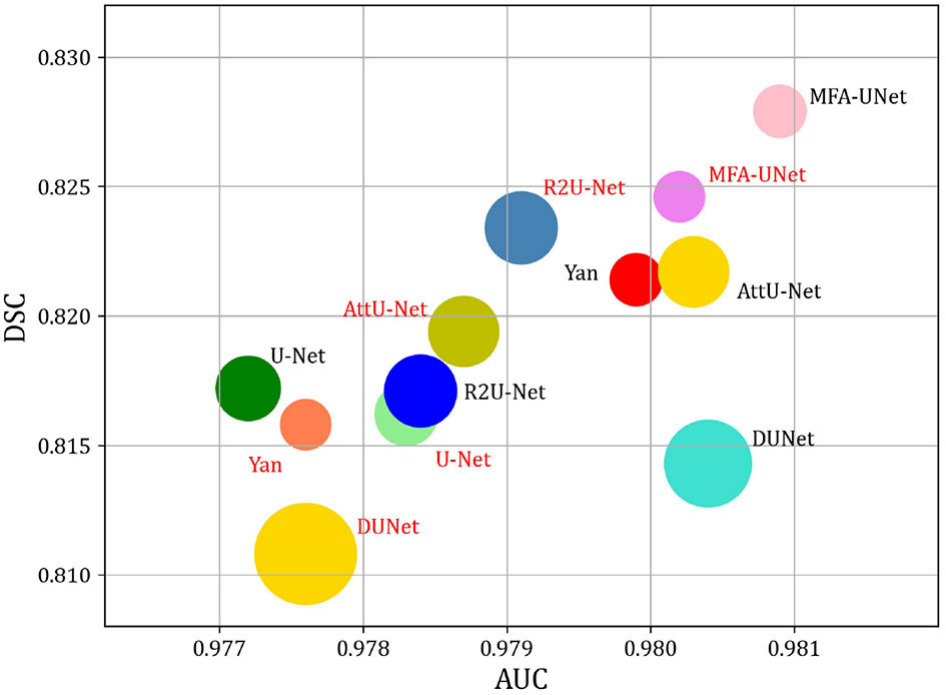
Comparison of model Params and performance. Note that the size of the circle indicates the number of model parameters. DSC, and AUC are used to evaluate the performance of models. The red font indicates that the method utilizes the whole image as input, while the black font indicates that the method utilizes patches as input.

We trained MFA-UNet and other methods separately on image patches and whole images, subsequently evaluating them to obtain various metrics for the performance comparison of the mentioned methods. The metrics used encompass the number of trainable parameters in the model, Floating Point Operations PerSecond (FLOPs), inference time for a single image, DSC, and AUC. Table 5 provides an overview of the trainable parameters, FLOPs, and inference time for MFA-UNet and other methods. To visually demonstrate the performance of each model in terms of trainable parameters, DSC, and AUC, we present a ball chart in Figure 10. According to Table 5, UNet has approximately 3.45 million trainable parameters. After incorporating all the proposed modules into UNet, MFA-UNet achieves smaller trainable parameters while maintaining excellent segmentation accuracy. In comparison, DUNet has 7.41 million trainable parameters but exhibits weaker performance in terms of DSC. Additionally, Attention U-Net and R2U-Net, as variants of U-Net, have a similar number of parameters to UNet. Attention U-Net, with the inclusion of the attention mechanism, achieves high segmentation accuracy, validating the effectiveness of this module. Our MFA-UNet outperforms other methods in terms of DSC and AUC, while maintaining smaller model parameters and complexity.

**Table 5:** Comparison of parameters (unit: M), FLOPs (unit: G), inference time for models with patch segmentation and panoramic segmentation on a single image (unit: s) among different methods on the DRIVE dataset.

**Method**	**Parameters (M)**	**FLOPs (G)**	**Inference time (s)**	
			**Patch**	**Whole image**
U-Net Ronneberger et al. (2015)	3.45	0.05	0.211	0.218
R2U-Net Alom et al. (2018)	3.91	0.12	0.506	0.332
AttU-Net Oktay et al. (2018)	3.49	0.06	0.311	0.227
Yan et al. (2019)	2.56	0.93	1.213	1.1
DUNet Jin et al. (2019)	7.41	0.23	0.765	0.476
Proposed	2.39	0.12	0.399	0.332

When we use whole images as training data, all compared methods exhibit varying degrees of reduction in DSC and AUC, with the exception of UNet and R2U-Net. We attribute this decline to the fact that when using entire images as input, segmentation models may not adequately focus on the smaller, finer blood vessels, thereby resulting in incomplete vessel segmentation and decreased performance. Inference time is reduced compared to patch-based methods, as utilizing an overlapping patch strategy increases the number of image samples significantly. Patch-based methods require segmentation of more images, extending inference time. Nevertheless, this approach enhances the segmentation performance of different models and meets the high-precision requirements of tasks like retinal vessel segmentation.

## 6 Discussion

Figure 9 shows that the incorporation of PAM into the decoder restores the structure of some of the macrovessels, while the addition of MSAM empowers MFA-UNet to accurately segment the intricate branching patterns of microvessels. To adjust the weight of the features within the skip path, we propose MSAM based on the self-attention mechanism and introduce it into the skip path of MFA-UNet. Unlike traditional convolutional neural networks, where the convolutional layer integrates the local feature information of the image through windowed convolution operations, resulting in a network with a restricted perceptual field, MSAM has a self-attention mechanism with a global perceptual field. This empowers MFA-UNet to effectively capture the interdependencies between vessel pixels and other pixels within the image.

The effectiveness of the multi-objective segmentation strategy is demonstrated by the improvement observed across all metrics in Table 2. Since a single network is inadequate for segmenting both macrovessels and microvessels, we established multiple branches in the decoder, each with different optimization objectives. This structure introduces deep supervision and enables the segmentation of macrovessels and microvessels to be separate, allowing us to adjust the optimization objectives of MFA-UNet. The primary structure of the model is utilized for feature extraction and recovery of the rough vessel structure. The feature maps are subsequently used for the reconstruction of vessels with different widths in MBDM and the merging of macrovessels and microvessels, thereby preserving more vessel structures in the final result. The visualization results in Figure 9 also prove our thinking. It is notable that the time to train a single epoch of MFA-UNet increases from 124 to 147 s after adding branch 1 and branch 2, such a design improves the segmentation performance but without increasing the complexity of the model.

In our study, we investigated the impact of the position of the attention mechanism within the model. When the attention mechanism is applied after the cascade of feature maps from the decoder and skip path, we observed an improvement in sensitivity but a decline in specificity. Conversely, when the attention mechanism is applied after the cascade fusion of feature maps and upsampling, we observed an increase in specificity but a decrease in sensitivity. We hypothesize that adjusting the feature map within the skip path can enhance the vessel features in the feature map, but the model is prone to misclassify background pixels as vessel pixels. On the other hand, adjusting the upsampled feature maps will make the model focus on the classification of background pixels. Considering the requirement for a balanced performance across multiple metrics, we incorporate MSA into the skip path to enable the decoder to utilize multi-scale features for better segmentation. Additionally, we position the attention mechanism after the convolutional layer of the decoder to mitigate the misclassification rate of the model.

In comparison to the DRIVE, STARE, HRF, IOSTAR, and FIVES datasets, the CHASE-DB1 dataset is specifically curated from the eyes of children to mitigate the interference caused by lesions associated with eye diseases. Figure 7 illustrates that during the testing phase, the segmentation of the optic disc boundary by MFA-UNet resulted in a low specificity improvement of the model. We attribute this observation to the high contrast exhibited by the optic disc in the fundus image of the CHASE-DB1 dataset, as well as the similarity in shape between the optic disc boundary and blood vessels. Consequently, the model tends to misclassify the optic disc boundary as blood vessels.

In the IOSTAR dataset, We observed a common issue among all segmentation models evaluated on this dataset: square-shaped convolutional kernels struggle to preserve the curvatures of blood vessels, particularly those with smaller curvatures. We believe that during the encoding process, the perception of blood vessels of model with larger curvatures is compromised due to their relatively low proportion in the overall structure. Additionally, the local feature extraction capabilities of the convolutional kernels fail to accurately segment distributed and highly curved vessel structures.

In the FIVES dataset, our proposed model has achieved optimal performance across all metrics. We visualized the segmentation results of MFA-UNet and UNet on glaucoma and DR images to demonstrate the influence of retinal image characteristics on segmentation performance (Figure 7). The left column in Figure 7 shows the segmentation results on glaucoma images, while the right column displays the results on DR images. We observed that the low contrast and intensity in glaucoma images significantly degraded the segmentation performance of UNet. However, MFA-UNet partially restored the interrupted vessels using self-attention mechanisms, although the segmentation results were still affected. Furthermore, the leopard-like appearance present in glaucoma images, resembling blood vessels, caused MFA-UNet to misclassify background pixels as vessel pixels. In contrast, DR images exhibited higher intensity and contrast, enabling MFA-UNet to accurately segment most vessel structures. These observations are further supported by the quantitative results presented in Table 2.

The proposed MFA-UNet has achieved competitive performance on multiple public datasets, yet there are still some limitations worth discussing. Convolutional neural networks (CNNs) inherently involve downsampling to reduce the dimensionality of image information, which can result in the loss of fine image details. Consequently, CNN-based methods may face challenges in accurately segmenting fine blood vessels, even when incorporating multi-scale feature information during the decoding process. To address this limitation, we believe that leveraging the information from the original image to refine the segmentation mask can further enhance the sensitivity and DSC of the segmentation framework. Recent studies have shown promising results by employing post-processing techniques such as dense conditional random fields (Lin et al., 2019), morphological reconstruction (Soomro et al., 2019), and probability-regularized random walks (Mou et al., 2020).

In addition, it is important to note that the proposed approach includes preprocessing the input of MFA-UNet to enhance the contrast of blood vessels, which contributes to improved segmentation performance. Consequently, the results may not be optimal when conducting segmentation on unprocessed images. Furthermore, while our method has been evaluated on datasets encompassing various categories of ocular diseases, the performance of MFA-UNet has not been specifically validated on a single ocular disease dataset that exhibits varying severity levels. As a future direction, we are considering the utilization of graph convolutional neural networks to comprehensively analyze the vascular skeleton. This approach would enable the establishment of dependencies between vascular nodes and endpoints, facilitating the capture of contextual information and ultimately leading to more accurate vascular segmentation.

## 7 Conclusion

In this study, we present MFA-UNet, a novel neural network architecture that leverages self-attention mechanisms and multi-branch decoding modules to enhance the accuracy of microvascular segmentation and preserve microvessel structure. We also adopt preprocessing techniques to improve the quality of fundus images obtained by the fundus camera and employ patch-based data augmentation methods to mitigate overfitting issues that may arise due to the limited number of training samples. The MSAM performs the fusion of multi-scale features and establishes inter-pixel dependencies to enable the model with a global perceptual field and improve the segmentation performance on microvessels. Additionally, the MBDM enables the model to segment macrovessels and microvessels separately and merge the segmentation results to obtain an excellent segmentation mask, resulting in better performance in the segmentation of both macrovessels and microvessels. The PAM is included in the decoder to reduce the misclassification rate of the model. The experimental results show that the MFA-UNet has excellent performance in retinal vessel segmentation and outperforms current state-of-the-art algorithms in several metrics on the DRIVE, STARE, CHASEDB1, HRF, IOSTAR, and FIVES datasets. Moreover, MFA-UNet has smaller model parameters and complexity, requiring only 0.399 s to segment an image, suggesting that the proposed method holds promise for being transplanted into embedded software, thus further advancing the intelligence level of fundus cameras in the realm of vessel segmentation.

## Data availability statement

The datasets [DRIVE] for this study can be found in the [DRIVE: Digital Retinal Images for Vessel Extraction] [https://drive.grand-challenge.org/]. The datasets [STARE] for this study can be found in the [STARE: STructured Analysis of the Retina] [https://cecas.clemson.edu/~ahoover/stare/probing/index.html]. The datasets [CHASE_DB1] for this study can be found in the [Retinal Image Analysis] [https://blogs.kingston.ac.uk/retinal/chasedb1/]. The datasets [HRF] for this study can be found in the [High-Resolution Fundus (HRF) Image Database] [https://www5.cs.fau.de/research/data/fundus-images/]. The datasets [IOSTAR] for this study can be found in the [IOSTAR Retinal Vessel Segmentation Dataset] [https://www.idiap.ch/software/bob/docs/bob/bob.db.iostar/stable/]. The datasets [FIVES] for this study can be found in the [FIVES: A Fundus Image Dataset for Artificial Intelligence based Vessel Segmentation] [https://figshare.com/articles/figure/FIVES_A_Fundus_Image_Dataset_for_AI-based_Vessel_Segmentation/19688169].

## Author contributions

JCa, JCh, YG, and JL were responsible for the initial plan and study design. JCh and JL collected the data. JCa and JCh performed the experiments, analyzed the data, and wrote the paper. JCa and YG revised the papers. All authors contributed to the article and approved the submitted version.

## References

[B1] Alom M. Z. Hasan M. Yakopcic C. Taha T. M. Asari V. K. (2018). Recurrent residual convolutional neural network based on U-net (R2U-Net) for medical image segmentation. arXiv. 10.48550/arXiv.1802.06955

[B2] Badawi S. A. Fraz M. M. Shehzad M. Mahmood I. Javed S. Mosalam E.. (2022). Detection and grading of hypertensive retinopathy using vessels tortuosity and arteriovenous ratio. J. Digit. Imaging 35, 281–301. 10.1007/s10278-021-00545-z 35013827 PMC8921404

[B3] Barkana B. D. Saricicek I. Yildirim B. (2017). Performance analysis of descriptive statistical features in retinal vessel segmentation via fuzzy logic, ANN, SVM, and classifier fusion. Knowl.-Based Syst. 118, 165–176. 10.1016/j.knosys.2016.11.022

[B4] Cao Y. Liu L. Chen X. Man Z. Lin Q. Zeng X.. (2023). Segmentation of lung cancer-caused metastatic lesions in bone scan images using self-defined model with deep supervision. Biomed. Signal Process. Control 79, 104068. 10.1016/j.bspc.2022.104068

[B5] Chen X. Wang X. Zhang K. Fung K.-M. Thai T. C. Moore K.. (2022). Recent advances and clinical applications of deep learning in medical image analysis. Med. Image Anal. 79, 102444. 10.1016/j.media.2022.102444 35472844 PMC9156578

[B6] Deng X. Ye J. (2022). A retinal blood vessel segmentation based on improved d-mnet and pulse-coupled neural network. Biomed. Signal Process. Control 73, 103467. 10.1016/j.bspc.2021.103467

[B7] Feng S. Zhuo Z. Pan D. Tian Q. (2020). CcNet: a cross-connected convolutional network for segmenting retinal vessels using multi-scale features. Neurocomputing 392, 268–276. 10.1016/j.neucom.2018.10.098

[B8] Garg S. Sivaswamy J. Chandra S. (2007).“Unsupervised curvature-based retinal vessel segmentation,” in *2007 4th IEEE International Symposium on Biomedical Imaging: From Nano to Macro* (Arlington, VA: IEEE), 344–347. 10.1109/ISBI.2007.356859

[B9] Gegundez-Arias M. E. Marin-Santos D. Perez-Borrero I. Vasallo-Vazquez M. J. (2021). A new deep learning method for blood vessel segmentation in retinal images based on convolutional kernels and modified U-net model. Comput. Methods Programs Biomed. 205, 106081. 10.1016/j.cmpb.2021.106081 33882418

[B10] Guo S. Wang K. Kang H. Zhang Y. Gao Y. Li T.. (2019). BTS-DSN: deeply supervised neural network with short connections for retinal vessel segmentation. Int. J. Med. Inform. 126, 105–113. 10.1016/j.ijmedinf.2019.03.015 31029251

[B11] Guo X. Chen C. Lu Y. Meng K. Chen H. Zhou K.. (2020). Retinal vessel segmentation combined with generative adversarial networks and dense U-net. IEEE Access 8, 194551–194560. 10.1109/ACCESS.2020.3033273

[B12] He K. Lian C. Zhang B. Zhang X. Cao X. Nie D.. (2021). HF-UNet: learning hierarchically inter-task relevance in multi-task U-net for accurate prostate segmentation in CT images. IEEE Trans. Med. Imaging 40, 2118–2128. 10.1109/TMI.2021.3072956 33848243

[B13] Hoover A. Kouznetsova V. Goldbaum M. (2000). Locating blood vessels in retinal images by piecewise threshold probing of a matched filter response. IEEE Trans. Med. Imaging 19, 203–210. 10.1109/42.845178 10875704

[B14] Jiang Y. Wang F. Gao J. Liu W. (2020). Efficient BFCN for automatic retinal vessel segmentation. J. Ophthalmol. 2020, 6439407. 10.1155/2020/6439407 33489334 PMC7803293

[B15] Jiang Y. Zhang H. Tan N. Chen L. (2019). Automatic retinal blood vessel segmentation based on fully convolutional neural networks. Symmetry 11, 1112. 10.3390/sym11091112

[B16] Jiang Z. Yepez J. An S. Ko S. (2017). Fast, accurate and robust retinal vessel segmentation system. Biocybern. Biomed. Eng. 37, 412–421. 10.1016/j.bbe.2017.04.001

[B17] Jin K. Huang X. Zhou J. Li Y. Yan Y. Sun Y.. (2022). FIVES: a fundus image dataset for artificial intelligence based vessel segmentation. Sci. Data 9, 475. 10.1038/s41597-022-01564-3 35927290 PMC9352679

[B18] Jin Q. Meng Z. Pham T. D. Chen Q. Wei L. Su R.. (2019). DUNet: a deformable network for retinal vessel segmentation. Knowl.-Based Syst. 178, 149–162. 10.1016/j.knosys.2019.04.025

[B19] Kamran S. A. Hossain K. F. Tavakkoli A. Zuckerbrod S. L. Sanders K. M. Baker S. A.. (2021). RV-GAN: segmenting retinal vascular structure in fundus photographs using a novel multi-scale generative adversarial network. MICCAI 12908, 34–44. 10.1007/978-3-030-87237-3_4

[B20] Kande G. B. Subbaiah P. V. Savithri T. S. (2010). Unsupervised fuzzy based vessel segmentation in pathological digital fundus images. J. Med. Syst. 34, 849–858. 10.1007/s10916-009-9299-0 20703624

[B21] Kar M. K. Neog D. R. Nath M. K. (2023). Retinal vessel segmentation using multi-scale residual convolutional neural network (MSR-net) combined with generative adversarial networks. Circuits Syst. Signal Process 42, 1206–1235. 10.1007/s00034-022-02190-5

[B22] Lee C.-Y. Xie S. Gallagher P. Zhang Z. Tu Z. (2014). Deeply-supervised nets. arXiv. 10.48550/arXiv.1409.5185

[B23] Li Q. Feng B. Xie L. Liang P. Zhang H. Wang T.. (2016). A cross-modality learning approach for vessel segmentation in retinal images. IEEE Trans. Med. Imaging, 35, 109–118. 10.1109/TMI.2015.2457891 26208306

[B24] Lin A. Chen B. Xu J. Zhang Z. Lu G. Zhang D.. (2022). Ds-transunet: dual swin transformer u-net for medical image segmentation. IEEE Trans. Instrum. Meas. 71, 1–15. 10.1109/TIM.2022.3178991

[B25] Lin Y. Zhang H. Hu G. (2019). Automatic retinal vessel segmentation via deeply supervised and smoothly regularized network. IEEE Access 7, 57717–57724. 10.1109/ACCESS.2018.2844861

[B26] Lin Z. Huang J. Chen Y. Zhang X. Zhao W. Li Y.. (2021). A high resolution representation network with multi-path scale for retinal vessel segmentation. Comput. Methods Programs Biomed. 208, 106206. 10.1016/j.cmpb.2021.106206 34146772

[B27] Liu C. Gu P. Xiao Z. (2022a). Multiscale U-net with spatial positional attention for retinal vessel segmentation. J. Healthc. Eng. 2022, 5188362. 10.1155/2022/5188362 35047151 PMC8763561

[B28] Liu D. Wang L. Du Y. Cong M. Li Y. (2022b). 3-D prostate mr and trus images detection and segmentation for puncture biopsy. IEEE Trans. Instrum. Meas. 71, 1–13. 10.1109/TIM.2022.3192292

[B29] Liu X. Bai Z. Li Q. (2019). ‘On retinal vessel segmentation using FCN,” in *2019 IEEE International Conference on Signal, Information and Data Processing (ICSIDP)* (Chongqing: IEEE), 1–6. 10.1109/ICSIDP47821.2019.9173099

[B30] Liu Y. Shen J. Yang L. Bian G. Yu H. (2023). Resdo-unet: a deep residual network for accurate retinal vessel segmentation from fundus images. Biomed. Signal Process. Control 79, 104087. 10.1016/j.bspc.2022.104087

[B31] Lu X. Shao F. Xiong Y. Yang Y. (2020). Retinal vessel segmentation method based on two-stream networks. Acta Optica Sinica 40, 47–55. 10.3788/AOS202040.0410002

[B32] Mahapatra S. Agrawal S. Mishro P. K. Pachori R. B. (2022). A novel framework for retinal vessel segmentation using optimal improved frangi filter and adaptive weighted spatial FCM. Comput. Biol. Med. 147, 105770. 10.1016/j.compbiomed.2022.105770 35767920

[B33] Mazlan A. U. Sahabudin N. A. Remli M. A. Ismail N. S. N. Mohamad M. S. Nies H. W.. (2021). A review on recent progress in machine learning and deep learning methods for cancer classification on gene expression data. Processes 9, 1466. 10.3390/pr9081466

[B34] Milletari F. Navab N. Ahmadi S.-A. (2016). “V-Net: fully convolutional neural networks for volumetric medical image segmentation,” in 2016 Fourth International Conference on 3D Vision (3DV) (Stanford, CA: IEEE), 565–571. 10.1109/3DV.2016.79

[B35] Miotto R. Wang F. Wang S. Jiang X. Dudley J. T. (2017). Deep learning for fealthcare: review, opportunities and challenges. Brief. Bioinform. 19, 1236–1246. 10.1093/bib/bbx044 28481991 PMC6455466

[B36] Mo J. Zhang L. (2017). Multi-level deep supervised networks for retinal vessel segmentation. Int. J. Comput. Assist. Radiol. Surg. 12, 2181–2193. 10.1007/s11548-017-1619-0 28577175

[B37] Monemian M. Rabbani H. (2021). Analysis of a novel segmentation algorithm for optical coherence tomography images based on pixels intensity correlations. IEEE Trans. Instrum. Meas. 70, 1–12. 10.1109/TIM.2020.301703733776080

[B38] Mou L. Chen L. Cheng J. Gu Z. Zhao Y. Liu J.. (2020). Dense dilated network with probability regularized walk for vessel detection. IEEE Trans. Med. Imaging 39, 1392–1403. 10.1109/TMI.2019.2950051 31675323

[B39] Naveed K. Abdullah F. Madni H. A. Khan M. A. Khan T. M. Naqvi S. S.. (2021). Towards automated eye diagnosis: an improved retinal vessel segmentation framework using ensemble block matching 3D filter. Diagnostics 11, 114. 10.3390/diagnostics11010114 33445723 PMC7828181

[B40] Odstrcilík J. Kolář R. Budai A. Hornegger J. Jan J. Gazárek J.. (2013). Retinal vessel segmentation by improved matched filtering: evaluation on a new high-resolution fundus image database. IET Image Process. 7, 373–383. 10.1049/iet-ipr.2012.0455

[B41] Oktay O. Schlemper J. Folgoc L. Lee M. Heinrich M. Misawa K.. (2018). Attention U-net: learning where to look for the pancreas. arXiv. 10.48550/arXiv.1804.03999

[B42] Ouyang J. Liu S. Peng H. Garg H. Thanh D. N. H. (2023). *Lea* U-net: a U-net-based deep learning framework with local feature enhancement and attention for retinal vessel segmentation. Complex Intell. Syst. 9, 6753–6766. 10.1007/s40747-023-01095-3

[B43] Owen C. G. Rudnicka A. R. Mullen R. Barman S. A. Monekosso D. N. Whincup P. H.. (2009). Measuring retinal vessel tortuosity in 10-year-old children: validation of the computer-assisted image analysis of the retina (CAIAR) program. Investig. Ophthalmol. Vis. Sci. 50, 2004–2010. 10.1167/iovs.08-3018 19324866

[B44] Palanivel D. A. Natarajan S. Gopalakrishnan S. (2020). Retinal vessel segmentation using multifractal characterization. Appl. Soft Comput. 94, 106439. 10.1016/j.asoc.2020.106439

[B45] Pan J. Gong J. Yu M. Zhang J. Guo Y. Zhang G.. (2022). A multilevel remote relational modeling network for accurate segmentation of fundus blood vessels. IEEE Trans. Instrum. Meas. 71, 1–14. 10.1109/TIM.2022.3203114

[B46] Pang S. Du A. Yu Z. Orgun M. A. (2021). 2D medical image segmentation via learning multi-scale contextual dependencies. Methods 202, 40–53. 10.1016/j.ymeth.2021.05.015 34029714

[B47] Ricci E. Perfetti R. (2007). Retinal blood vessel segmentation using line operators and support vector classification. IEEE Trans. Med. Imaging 26, 1357–1365. 10.1109/TMI.2007.898551 17948726

[B48] Ronneberger O. Fischer P. Brox T. (2015). “U-Net: convolutional networks for biomedical image segmentation,” in Medical Image Computing and Computer-Assisted Intervention-MICCAI 2015, eds N. Navab, J. Hornegger, W. M. Wells, and A. F. Frangi (Cham: Springer International Publishing), 234–241. 10.1007/978-3-319-24574-4_28

[B49] Samuel P. M. Veeramalai T. (2021). VSSC net: vessel specific skip chain convolutional network for blood vessel segmentation. Comput. Methods Programs Biomed. 198, 105769. 10.1016/j.cmpb.2020.105769 33039919

[B50] Saranya P. Prabakaran S. Kumar R. Das E. (2022). Blood cessel segmentation in retinal fundus images for proliferative diabetic retinopathy screening using deep learning. Vis. Comput. 38, 977–992. 10.1007/s00371-021-02062-0

[B51] Shen X. Xu J. Jia H. Fan P. Dong F. Yu B.. (2022). Self-attentional microvessel segmentation via squeeze-excitation transformer U-net. Comput. Med. Imaging Graph. 97, 102055. 10.1016/j.compmedimag.2022.102055 35320771

[B52] Simonyan K. Zisserman A. (2014). Very deep convolutional networks for large-scale image recognition. arXiv. 10.48550/arXiv.1409.1556

[B53] Soomro T. A. Afifi A. J. Ali Shah A. Soomro S. Baloch G. A. Zheng L.. (2019). Impact of image enhancement technique on CNN model for retinal blood vessels segmentation. IEEE Access 7, 158183–158197. 10.1109/ACCESS.2019.2950228

[B54] Su Y. Cheng J. Cao G. Liu H. (2022). How to design a deep neural network for retinal vessel segmentation: an empirical study. Biomed. Signal Process. Control 77, 103761. 10.1016/j.bspc.2022.103761

[B55] Szegedy C. Liu W. Jia Y. Sermanet P. Reed S. Anguelov D.. (2015). “Going deeper with convolutions,” in 2015 IEEE Conference on Computer Vision and Pattern Recognition (CVPR) (Boston, MA: IEEE), 1–9. 10.1109/CVPR.2015.7298594

[B56] Wang C. Zhao Z. Yi Y. (2021). Fine retinal vessel segmentation by combining nest U-net and patch-learning. Soft Comput. 25, 1–14. 10.1007/s00500-020-05552-w

[B57] Wang D. Haytham A. Pottenburgh J. Saeedi O. Tao Y. (2020). Hard attention net for automatic retinal vessel segmentation. IEEE J. Biomed. Health Inform. 24, 3384–3396. 10.1109/JBHI.2020.3002985 32750941

[B58] Wang X. Jiang X. Ren J. (2019). Blood vessel segmentation from fundus image by a cascade classification framework. Pattern Recognit. 88, 331–341. 10.1016/j.patcog.2018.11.030

[B59] Woo S. Park J. Lee J.-Y. Kweon I.-S. (2018). “CBAM: convolutional block attention module,” in Computer Vision–*ECCV 2018: 15th European Conference, Munich, Germany, September 8–14* (New York, NY: ACM), 3–19. 10.1007/978-3-030-01234-2_1

[B60] Wu Y. Xia Y. Song Y. Zhang Y. Cai W. (2018). “Multiscale network followed network model for retinal vessel segmentation,” in Medical Image Computing and Computer Assisted Intervention-MICCAI 2018, eds A. F. Frangi, J. A. Schnabel, C. Davatzikos, C. Alberola-López, and G. Fichtinger (Cham: Springer International Publishing), 119–126. 10.1007/978-3-030-00934-2_14

[B61] Yan Z. Yang X. Cheng K. (2019). A three-stage deep learning model for accurate retinal vessel segmentation. IEEE J. Biomed. Health Inform. 23, 1427–1436. 10.1109/JBHI.2018.2872813 30281503

[B62] Yan Z. Yang X. Cheng K.-T. (2018). Joint segment-level and pixel-wise losses for deep learning based retinal vessel segmentation. IEEE Trans. Biomed. Eng. 65, 1912–1923. 10.1109/TBME.2018.2828137 29993396

[B63] Zhang J. Dashtbozorg B. Bekkers E. Pluim J. P. W. Duits R. ter Haar Romeny B. M. (2016). Robust retinal vessel segmentation via locally adaptive derivative frames in orientation scores. IEEE Trans. Med. Imaging 35, 2631–2644. 10.1109/TMI.2016.2587062 27514039

[B64] Zhao R. Li Q. Wu J. You J. (2021). A nested u-shape network with multi-scale upsample attention for robust retinal vascular segmentation. Pattern Recognit. 120, 107998. 10.1016/j.patcog.2021.107998

[B65] Zhou S. Nie D. Adeli E. Wei Q. Ren X. Liu X.. (2022). Semantic instance segmentation with discriminative deep supervision for medical images. Med. Image Anal. 82, 102626. 10.1016/j.media.2022.102626 36208573

